# Endophytic Fungi Natural Factories of Novel Bioactive Compounds

**DOI:** 10.1002/cbdv.71710

**Published:** 2026-09-15

**Authors:** Dilmurod Murodullayev, Jakhongir Movlanov, Liu Wei, Wei Liu, Maksud Isokov, Santosh Kumar Bose, Fazliddin Kobilov, Zamira Djumayeva, Buston Islamov, Jamila Sherkulova, Zafar Matyakubov, Muniskhon Meylieva, Munisa Ergasheva, Diyor Abduraxmanov, Guozheng Huang, Hongchen Jiang, Kakhramon Davranov, Guohui Pan, Nigora Rustamova

**Affiliations:** ^1^ Institute of Microbiology, Academy of Sciences of the Republic of Uzbekistan Tashkent Uzbekistan; ^2^ Karshi State University Karshi district Uzbekistan; ^3^ University of Geological Science, Center of Geoinnovation Technologies Tashkent Uzbekistan; ^4^ Qilu University of Technology (Shandong Academy of Sciences), Shandong Analysis and Test Center Jinan Shandong China; ^5^ Department of Horticulture Patuakhali Science and Technology University Dumki Patuakhali Bangladesh; ^6^ Samarkand State Pedagogical Institute Samarkand Uzbekistan; ^7^ Institute of Biochemistry, Samarkand State University University Boulevard 15 Samarkand Uzbekistan; ^8^ Khorezm Mamun Academy Khiva city, Khorezm Uzbekistan; ^9^ Institute of Hydrogeology and Engineering Geology University of Geological Science Tashkent Uzbekistan; ^10^ Samarkand State Medical University Samarkand Uzbekistan; ^11^ Department of Chemical Biology and Pharmaceutical Engineering School of Chemistry and Chemical Engineering, Anhui University of Technology Ma'anshan Anhui China; ^12^ School of Life Sciences Henan University Kaifeng China; ^13^ State Key Laboratory of Microbial Diversity and Innovative Utilization, Institute of Microbiology Chinese Academy of Sciences Beijing China

**Keywords:** biological activity, endophytic microorganism, natural product, specialized metabolites

## Abstract

Endophytic fungi represent a prolific and chemically diverse source of novel specialized metabolites with significant pharmacological potential. This systematic review examines 118 studies published between 2024 and 2025, reporting the discovery of 242 previously uncharacterized bioactive compounds. These metabolites belong to several major chemical classes, including peptides (**4**), terpenoids and steroids (**58**), alkaloids and other nitrogen‐containing compounds (**51**), polyketides (**85**), lactones (**22**), ketones (**5**), chromone compounds (**3**), and others (**9**). The genera *Aspergillus* (**28**), *Penicillium* (**21**), *Diaporthe* (**7**), *Talaromyces* (**10**), and *Trichoderma* (**6**) were identified as the most productive sources. The investigated endophytic fungi were isolated from a wide range of plant hosts and ecological niches, reflecting the extensive biodiversity of endophytic communities. Biological screening demonstrated that a substantial proportion of the tested compounds exhibited diverse bioactivities, including anti‐inflammatory, antibacterial, antifungal, antimicroalgal, and cytotoxic effects. Collectively, the remarkable structural diversity and broad biological profiles of metabolites derived from endophytic fungi highlight their strong potential as promising lead scaffolds for future pharmaceutical development.

## Graphical abstract

This review provides a systematic analysis of 242 natural products isolated from 42 genera of endophytic fungi, highlighting their remarkable structural diversity and broad spectrum of biological activities

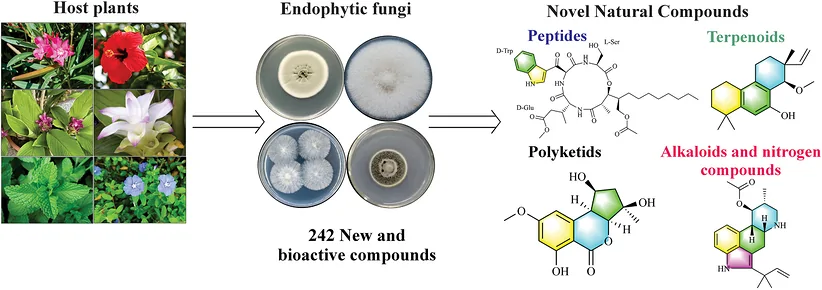

## 1 Introduction

Endophytic fungi colonize the internal tissues of healthy plants without causing apparent disease symptoms and are ubiquitous components of terrestrial ecosystems. These microorganisms have emerged as prolific producer of structurally diverse secondary metabolites and represent an important reservoir of biologically active natural products with significant pharmaceutical potential [1, 2, 3, 4, 5]. Their biosynthetic repertoire encompasses a broard range of chemical classes, including alkaloids, peptides, terpenoids, steroids, polyketides, phenolics, quinones, and flavonoids, many of which exhibit potent antimicrobial, anticancer, anti‐inflammatory, antioxidant, antiviral, enzyme‐inhibitory, and immunomodulatory activities [3, 6, 7, 8, 9, 10]. Due to their exceptional chemical diversity and biosynthetic versatility, endophytic fungi are increasingly recognized as promising sources of novel lead compounds for drug discovery and development. In addition to producing structurally unique metabolites, endophytic fungi exhibit remarkable metabolic plasticity, enabling the biotransformation of both natural and synthetic substrates into structurally modified derivatives with enhanced or altered biological properties [10]. This catalytic versatility significantly expands the accessible chemical space and facilitates the generation of novel bioactive molecules that are often challenging to obtain through conventional synthetic methods. Reflecting rapid progress in this field, Rustamova et al. reported 221 previously undescribed bioactive specialized metabolites isolated from endophytic fungi between 2018 and the present, underscoring the accelerating pace of metabolite discovery and structural characterization [11]. Among the diverse endophytic taxa studied, species belonging to the genera *Aspergillus* and *Penicillium* have received particular attention due to their exceptional biosynthetic capacity and consistent production of structurally unprecedented metabolates with diverse pharmacological activites [12, 13]. Over the past decade, numerous reviews have summarized specialized metabolites produced by endophytic fungi from various perspectives. Ojo et al. compiled 217 terpenoid metabolites isolated from plant‐associated fungi between 2019 and 2024, highlighting their structural diversity and broad spectrum of biological activities, including anti‐inflammatory, antimicrobial, cytotoxic, and enzyme‐inhibitory activities [14]. Zheng et al. summarized 449 specialized metabolites reported between 2017 and 2019 and discussed cultivation‐based strategies, particularly co‐culture approaches, for activating sryptic biosynthetic gene clusters and enhancing metabolite diversity [15]. Chen et al. reviewed 101 metabolites isolated from endophytic fungi associated with *Tripterygium wilfordii*, reporting their antibacterial, antifungal, antiviral, and cytotoxic properties [16]. Similarly, Adhikari et al. focused on anticancer metabolites derived from endophytic fungi associated with *Taxus* species, highlighting their potential for anticancer drug development [17]. Likewise, Cao et al. summarized the chemistry and biological activities of metabolites isolated fromendophytic fungi associated with *Huperzia serrata*, emphasizing their antimicrobial, anti‐inflammatory, cytotoxic, and neuroprotective activities [18]. More recently, Perumal et al. comprehensively evaluated the therapeutic potential of fungal endophyte‐derived metabolites, with particular emphasis on compounds exhibiting activity against clinically important microbial pathogens [19]. Recent advances have significantly expanded our understanding of microbial specialized metabolites beyond their traditional role as pharmaceutical lead compounds. Growing evidence suggests that these metabolites also serve as key regulators of host physiology, interspecies communication, and biological homeostasis. Singh et al. [20] described microbial specialized metabolites as central mediators of host regulatory networks, while Shah et al. [21] emphasized fungal endophytes as sustainable and renewable sources of structurally diverse bioactive natural products with considerable potential for future therapeutic development. Concurrently, progress in genome mining, multi‐omics integration, artificial intelligence‐assisted metabolite prediction, molecular networking, and synthetic biology has revolutionized natural product research by enabling the activation of previously silent biosynthetic gene clusters, accelerating metabolite dereplication, and facilitating the discovery of previously inaccessible chemical scaffolds from endophytic fungi [22]. The escalating global burden of antimicrobial resistance has heightened interest in endophytic fungi as alternative sources of anti‐infective agents. Numerous metabolites discussed in these review exhibit potent antibacterial and antifungal activities, offering structurally distinct molecular scaffolds that may overcome existing resistance mechanisms and support the development of next‐generation antimicrobial therapeutics [23].Endophyte‐derived metabolites exhibit a wide range of therapeutic activities, including anticancer, anti‐inflammatory, antioxidant, antiviral, and immunomodulatory effects. However, existing reviews primarily focus on specific host organisms, fungal groups, or classes of metabolites. This review offers an updated overview of recently discovered metabolites, highlighting their chemical diversity, biological activities, structure‐activity relationships, and future prospects for natural product discovery and drug development.

### 1.1 Methodology for Literature Selection

**FIGURE 1:**
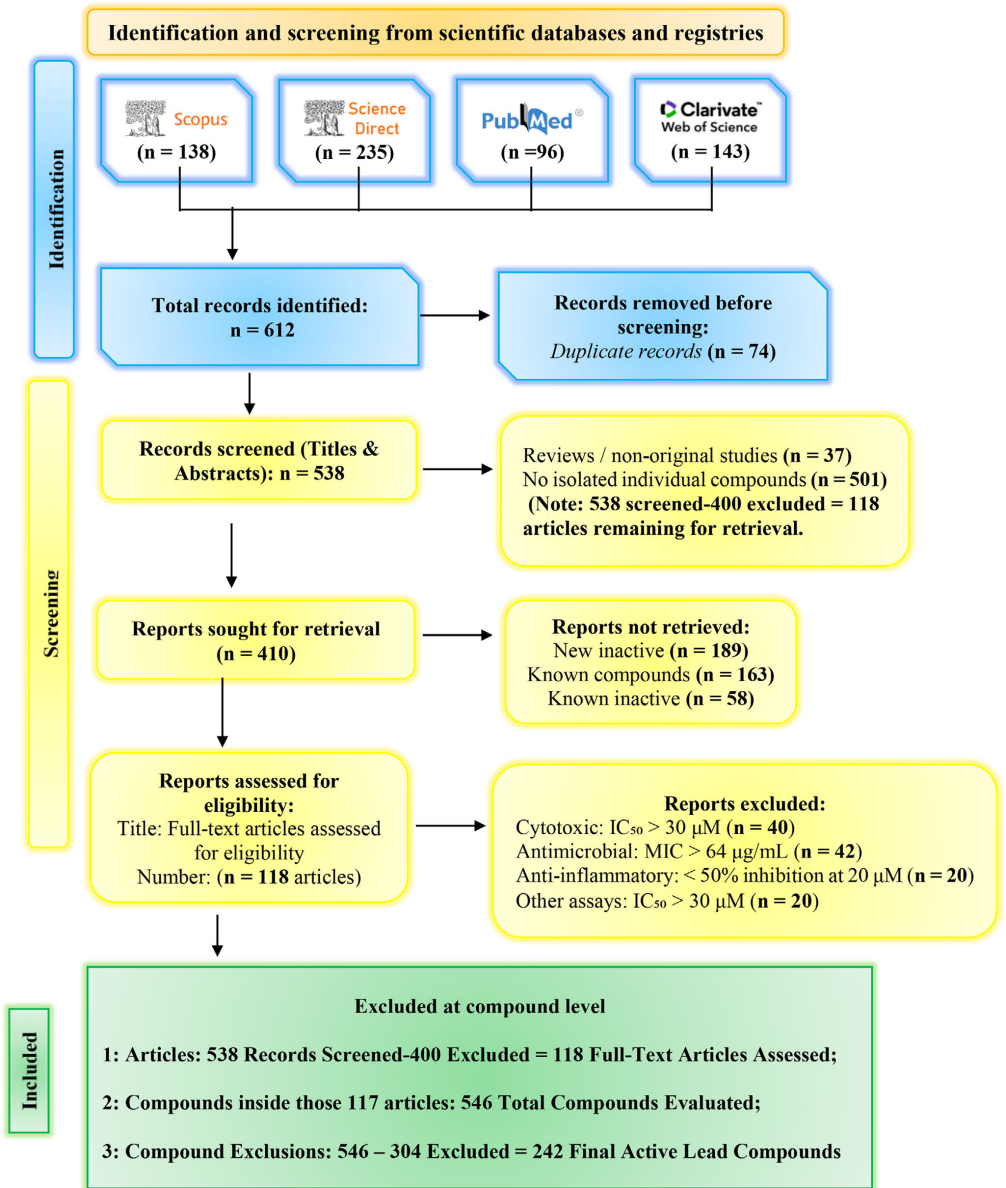
Literature selection of natural lead compounds derived from the endophytic fungi in this review.

**FIGURE 2:**
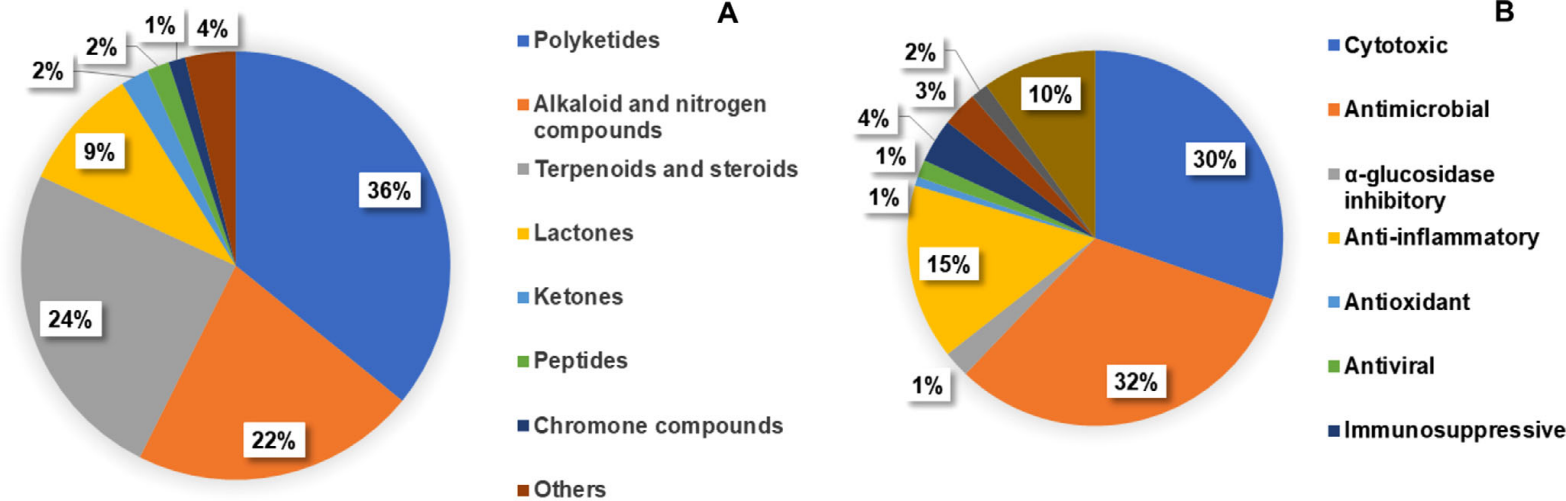
Classification (A) and biological properties (B) of the natural compounds, and relative abundance of the endophytic microorganism taxa from which these compounds were isolated.

**FIGURE 3:**
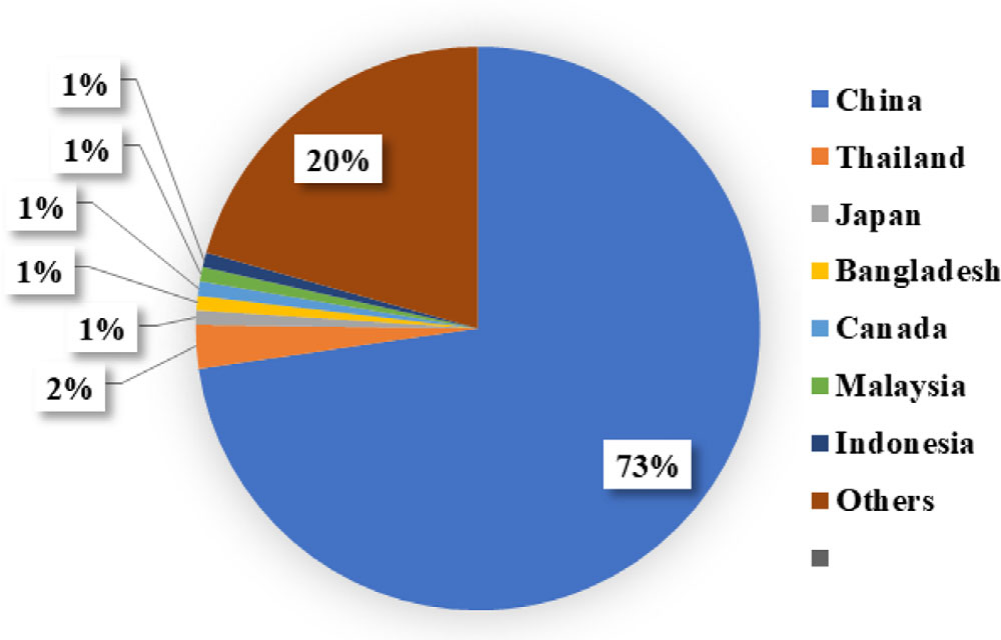
Geographic distribution of endophytic microorganism taxa from which the compounds were isolated.

**FIGURE 4:**
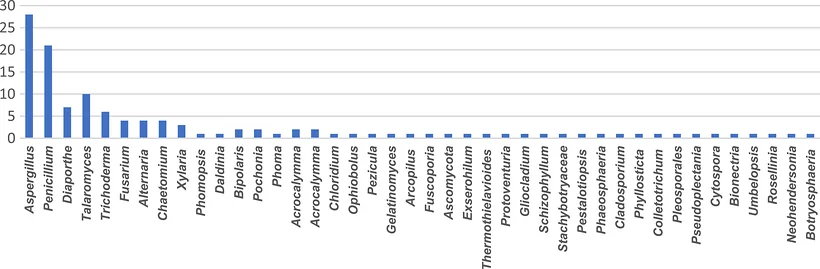
Number of endophytic microbial strains investigated in each genus.

This review was conducted in accordance with the preferred reporting items for systematic reviews and meta‐analyses (PRISMA) guidelines to ensure a transparent, reproducible, and systematic literature selection process (Figure 1). A comprehensive literature search was performed using the Scopus, ScienceDirect, PubMed, and Web of Science databases to identify relevant peer‐reviewed articles published between January 2024 and December 2025. The search strategy incorporated combinations of the keywords “endophyte,” endophytic fungi,″ endophytic fungus,″ and “novel secondary metabolites from endophytic fungi”. The initial search identified 386 publications. After removing irrelevant records and screening titles, abstracts, and full‐text articles according to predefined eligibility criteria, 117 studies were included in the qualitative analysis. This review focuses exclusively on structurally novel specialized metabolites isolated from plant‐associated endophytic fungi and includes only compounds with experimentally validated biological activities. Based on these selection criteria, 242 previously undescribed bioactive specialized metabolites were retained for comprehensive evaluation. The selected metabolites were systematically classified based on their geographical origin, producing fungal taxa, chemical classes, and reported biological activities (Figures 2 and   3). Most compounds were isolated from endophytic fungi originating in China, with species belonging to the genera *Aspergillus* and *Penicillium* representing the predominant producers of bioactive metabolites (Figure 4). Chemically, polyketides and terpenoids were the most abundant metabolite classes, while antimicrobial and cytotoxic activities were the most frequently reported pharmacological properties (Figures 3 and 4). Collectively, these findings highlight the remarkable structural diversity and significant pharmaceutical potential of specialized metabolites derived from endophytic fungi (Table 1). Studies were excluded if they met one or more of the following criteria: (i) the reported metabolites lacked structural novelty; (ii) biological activities were not experimentally validated; or (iii) the compounds exhibited weak, negligible, or statistically insignificant bioactivity. To ensure consistency and emphasize pharmacologically relevant metabolites, quantitative inclusion thresholds were established based on widely accepted standards in natural products and pharmacological research. Compounds were considered eligible if they satisfied at least one of the following criteria: Cytotoxic activity (IC_50_ < 30 µM), antimicrobial activity (MIC ≤ 64 µg/mL), anti‐inflammatory activity (>50% inhibition at 20 µM), antioxidant activity (IC_50_ ≤ 50 µM), or antimicroalgal activity (IC_50_ ≤ 30 µM). For biological activities lacking universally accepted quantitative benchmarks, eligibility was determined according to the evaluation criteria reported in the original studies and supported by relevant literature. Compounds that exceeded these predefined thresholds or lacked quantitative bioactivity data were excluded from further analysis. The application of rigorous selection criteria produced a high‐quality dataset consisting of structurally novel and pharmacologically relevant specialized metabolites derived from endophytic fungi. This curated dataset offers a solid foundation for critically evaluating recent advances in endophytic fungal natural products, identifying emerging structure‐activity relationships, and highlighting promising lead compounds for future applications in drug discovery, sustainable agriculture, and industrial biotechnology.

**TABLE 1:** Comprehensive summary of natural products and their pharmacological activities discussed in this review.

Strain	Host plant	Collected country	Compound Name (No.)	Activity (with quantitative values)	References
Trichoderma lentiforme	Bruguiera gymnorrhiza	—	**1** and **2**	Cytotoxic IC_50 _= 0.76 ± 0.68 − 27.18 ± 3.60 µM and antimicrobial (MIC = 1.56 to 6.25 µM)	[1]
*Phaeosphaeria* sp.	*Conus literatus*	China	**3**	α‐glucosidase inhibitory (10 and 20 µM) and antiviral (15 and 20 µM)	[2]
*Aspergillus* sp. and *Penicillium* sp.	*Acanthus ilicifolius* L.,	China	**4**	Antimicrobial and cytotoxic (IC_50 _= 20.1 ± 0.9 µM)	[3]
Stachybotryaceae	Delphinium yunnanense	China	**5‐7**	Cytotoxic (IC_50 _= 1.80 ± 0.08 − 17.76 ± 0.97 µM)	[4]
Penicillium sp.	Sophora tonkinensis	China	**8**	Anti‐inflammatory (IC_50 _= 18.50 ± 1.26 µM)	[5]
*Talaromyces primulinus*	*Euphorbia sikkimensis*	China	**9**	Anti‐inflammatory (IC_50_ = 27.19 µM)	[6]
*Talaromyces* sp.	Mangrove	China	**10**	Anti‐inflammatory (IC_50_ = 11.33 µM)	[7]
*Diaporthe* sp.	*Rhododendron simsii* Planch.	China	**11‐**‐**15**	Immunosuppressive IC_50_ = 3.8−8.5 µM) and cytotoxic (IC_50_ = 9.6 ± 1.6 −26.71 µM)	[8]
Aspergillus sp.	Kandelia candel	China	**16**	Cytotoxic (IC_50_ = 26.71 µM)	[9]
Penicillium spinulosum	Emmenopterys henryi Oliv.	China	**17**–**21**	Antimicrobial (12.5‐50 µg/mL)	[10]
Fusarium equiseti	Catharanthus roseus (L.) G. Don	—	**22** and **23**	Antiproliferative (IC_50_ = 11.05 ± 0.28−24.87 ± 0.21 µM)	[11]
*Chaetomium nigricolor*	*Mahonia fortunei*	—	**24**	Neuroprotective (10 µg/mL)	[12]
Aspergillus sp.	Kandelia candel	China	**25**	Insecticidal (95%)	[13]
Penicillium oxalicum	Rhodomela confervoides	—	**26**	Antimicrobial (MIC = 4 and 2 µg/mL)	[14]
*Acrocalymma cycadis*,	*Sinomenium acutum*	—	**27**	Cytotoxic (IC_50_ = 2.89 ± 0.54−5.71 ± 1.22 µM	[15]
Diaporthe sp.	Etlingera littoralis	Thailand	**28**‐**30**	Antitubercular (MIC = 25‐50 µg/mL) and antimicrobial (MIC = 25‐50 µg/mL)	[16]
*Phyllosticta capitalensis*,	*Loropetalum chinense* var.	China	**31** and **32**	Anti‐inflammatory (IC_50_ = 21.65 ± 1.33 µM and 12.19 ± 1.08 µM)	[17]
*Colletotrichum camelliae*	*Camellia taliensis*	—	**33** and **34**	Antimicrobial (MIC = 31.25 µg/mL)	[18]
*Pleosporales* sp.	*Kandelia candel*	China	**35**‐**40**	Anti‐Inflammatory (IC_50_ = 19 and 25 µM)	[19]
*Talaromyces* sp.	*Kandelia obovata*	China	**41**–**44**	Anti‐Inflammatory (IC_50_ = 4.59−21.60 µM)	[20]
*Diaporthe phaseolorum*	*Ceriops tagal*	China	**45** and **46**	Immunosuppressive (IC_50_ = 10.831 ± 2.54−11.34 ± 0.67 µM)	[21]
*Penicillium bialowiezense*	*Euphorbia neriifolia*	China	**47**	Immunosuppressive (IC_50_ = 7.3 ± 0.8 µM and 6.3 ± 1.0 µM)	[22]
*Pseudoplectania* sp.	—	—	**47**–**50**	Cytotoxic (IC_50_ = 17.4 to 80.7 µM)	[23]
Penicillium macrosclerotiorum	Ilex pubescens Hook. et Arn.	—	**51**	Antimicrobial (MIC = 0.78 mg/mL)	[24]
Aspergillus sp.	Pseudostellaria heterophylla (Miq.)	China	**52**–**57**	Anti‐inflammatory (10 µM)	[25]
Aspergillus sp.	Zingiber officinale	China	**58**	Cytotoxic (IC_50_ = 0.40 µM)	[26].
Trichoderma sp.	Ganoderma lucidum	China	**59**	Anti‐inflammatory (20 µM)	[27]
*Penicillium fellutanum*	*Euphorbia helioscopia* L.	China	**60**	Cytotoxic (IC_50_ = 22.56 ± 2.30 and 18.15 ± 1.05 µM)	[28]
*Bipolaris maydis*		China	**61**	Cytotoxic (IC_50_ = 3.83 ± 0.16−7.94 ± 0.02 µM)	[29]
*Acrocalymma cycadis*,	*Sinomenium acutum*	China	**62**	Cytotoxic (IC_50_ = 27.02 ± 3.19 and 22.13 ± 0.17 µM)	[30]
Bionectria ochroleuca	Selaginella doederleinii	China	**63**	Antimicrobial (MIC = 64 µg/mL) and cytotoxic (IC_50_ = 20.27 and 25.84 µM)	[31]
Aspergillus sp.	Umbilicaria sp.	China	**64**	Agonistic (EC_50_ = 4.1 ± 0.3 µM)	[32]
*Alternaria porri* #25.	*Kandelia candel*	China	**65**	Cytotoxic (IC_50_ = 22.94 ± 0.53 µM)	[33]
Penicillium brefeldianum	Mangrove rhizosphere	China	**66** and **67**	Anti‐Inflammatory (20 µM)	[34]
Fusarium oxysporum	Salvia officinalis	China	**68**	Cytotoxic (IC_50_ = 10.15−25.34 µM) and antioxidant (IC_50_ = 15.43 ± 1.00 µg/mL and 30.23 ± 7.45 µg/mL)	[35]
Penicillium crustosum	Acanthus ilicifolius	China	**69**	Antimicrobial (MIC = 0.25 mg/mL)	[36]
Penicillium sizovae	Ferula sinkiangensis	China	**70** and **71**	Cytotoxic (IC_50_ = 14.2 ± 1.5−23.8 ± 0.7 µM)	[37]
Chaetomium globosum	Aquilaria crassna	Thailand	**72**	Cytotoxic (IC_50_ = 13.04 µM)	[38]
Penicillium restrictum	Psammosilene tunicoides	China	**73** and **74**	Antimicrobial (MIC = 1–32 µg/mL)	[39]
Penicillium oxalicum	Cedrus spp.	China	**75** and **76**	Cytotoxic (IC_50_ = 7.86 ± 0.81–29.65 ± 2.33 µM)	[40]
*Aspergillus* sp.	*Rhizophora stylosa*	Vietnam	**77** and **78**	Anti‐inflammatory (IC_50_ = 5.3 ± 0.2 and 20.5 ± 0.8 µM)	[41]
*Cladosporium* sp.	*Rubus tephrodes*	—	**79**	Antimicrobial (MIC = 8 and 16 µg/mL)	[42]
*Aspergillus taichungensis*	*Gelidium amansii*	China	**80**	Cytotoxic (IC_50_ = 1.7 ± 0.1‐23.4 ± 0.6 µM)	[43]
*Aspergillus puniceus*	Tobacco	China	**81** and **82**	Antimicrobial (15.4 ± 1.8–25.2 ± 1.6 mm)	[44]
*Aspergillus fumigatus*	Cigar‐tobacco	China	**83** and **84**	Antimicrobial (14.0 ± 1.8–22.4 ± 2.2 mm)	[45]
Aspergillus nidulans	Whitmania pigra	China	**85** and **86**	BuChE inhibitory (IC_50_ = 9.49 ± 0.51 and 2.03 ± 0.55 µM)	[46]
*Pochonia bulbillosa*	*Pyrrosia lingua*	China	**87**‐**89**	Immunosuppressive (EC_50_ = 13, 4.6, and 13 µM)	[47]
*Talaromyces amestolkiae*	*Kandelia obovata*	China	**90**	Cytotoxic (IC_50_ = 2.74 ± 0.20–4.58 ± 0.18 µM)	[48]
*Pseudeurotium bakeri*	*Macrocoma tenue subsp. sullivantii* Vitt.	China	**91**‐**98**	Cytotoxic (IC_50_ = 2.9 ± 0.3 to 27.8 ± 1.2 µM)	[49]
*Penicillium citrinum*	*Drynaria roosii*	China	**99** and **100**	Antimicrobial (MIC = 62.5 and 31.3 µM)	[50]
*Aspergillus alabamensis*	*Enhalus acoroides*	China	**101** and **102**	Antimicrobial (MIC = 10.0 µg/mL)	[51]
Xylaria arbuscula	Kandelia candel	China	**103**–**105**	Antimicrobial(MIC = 25 µM)	[52]
Diaporthe hongkongensis	Strychnos nux‐vomica	China	**106‐108**	Cytotoxic (IC_50_ = 1.35 and 3.59 µM)	[53]
*Rosellinia* sp.	*Glycyrrhiza inflata*	China	**109** and **111**	Cytotoxic (IC_50_ = 21.8 and 21.4 µM)	[54]
*Aspergillus spelaeus*	*Sophora tonkinensis*	China	**112** and **113**	Cytotoxic (IC_50_ = 3.9 ± 0.4 to 23 ± 1 µM)	[55]
Botryosphaeria dothidea	Liriodendron chinense	—	**114**‐**116**	Antimicrobial (MIC = 8 to 10 µg/mL) and anti‐neuroinflammatory (IC_50_ = 28.30 ± 3.08 µM)	[56]
Penicillium citrinum	Plantago asiatica	—	**117**	Osteogenic (2 µM)	[57]
Neohendersonia kickxii	Fagus crenata	Japan	**118**‐**120**	Cytotoxic (IC_50_ = 3.7−7.3 µM) and antimicrobial (MIC = 25 µg/mL)	[58]
*Phoma* sp.	*Gastrodia elata*	—	**121** and **122**	Antimicrobial (MIC = 64 and 8 µg/mL)	[59]
*Trichoderma koningiopsis*	*Rubia podantha*	China	**123**	Cytotoxic (IC_50_ = 8.09 µM)	[60]
*Talaromyces aculeatus*	*Aquilaria sinensis*	China	**124**	Anti‐inflammatory (IC_50_ = 24.17 ± 1.78 µM) and AChE inhibitory (IC_50_ = 18.43 µM)	[61]
*Cytospora heveae*	*Sonneratia caseolaris*	China	**125**	Cytotoxic (IC_50_ = 7.7 µM)	[62]
*Pestalotiopsis chamaeropis*	*Artemisia selengensis*	—	**126**	Anti‐inflammatory (IC_50_ = 19.7 ± 1.2 µM)	[63]
*Aspergillus* sp.	*Cerbera manghas*	China	**127**	Insecticidal (90.97%)	[64]
*Aspergillus japonicus*	*Nicotiana tabacum*	China	**128**	Herbicidal (IC_50_ = 21.5 µg/mL)	[65]
*Penicillium* sp.	*Rhododendron molle*	China	**129**	Antitumor (IC_50_ = 30.18 µM)	[66]
*Aspergillus versicolor*	*Ajuga multiflora*	China	**130**	Antimicrobial (MIC = 3.125 µg/mL)	[67]
*Chloridium virescens*	*Ganoderma lucidum*	China	**131** and **132**	Anti‐inflammatory (20 µM)	[68]
*Diaporthe* sp.	*S. acutum*	China	**133** and **134**	Antimicrobial (MIC = 64 µg/mL)	[69]
*Ophiobolus cirsii*	*Anaphalis lactea*	China	**135**‐**147**	Antitumor (20 µM)	[70]
Pochonia bulbillosa	Pyrrosia lingua	China	**148**–**154**	Cytotoxic (IC_50_ = 10.0 ± 0.5‐25.0 ± 1.0 µM) and immunosuppressive (EC_50_ = 6.3−27 µM)	[71]
Penicillium sp.	Macrozamia communis	—	**155**	Cytotoxic (IC_50_ = 8.3 µM) and antimicrobial (MIC =f 2.1 µg/mL)	[72]
Pezicula neosporulosa	Vaccinium dunalianum	China	**156**	Antimicrobial (MIC = 7.81 to 15.63 µg/mL)	[73]
Aspergillus sp.	Acanthus ilicifolius	China	**157**	AChE inhibitory (IC_50_ = 1.02 µM) and insecticidal (88%)	[74]
Gelatinomyces siamensis	Bambusa nutans	Thailand	**158**	Cytotoxic (IC_50_ = 11.31 ± 2.13 µg/mL)	[75]
Aspergillus niger	Camellia flavida	—	**159**	Cytotoxic (IC_50_ = 0.37 ± 0.06 µM and 2.04 ± 0.79 µM) and antimicrobial (27.1‐29.5 mm)	[76]
Aspergillus cristatus	Nicotiana tabacum	—	**160**	Antimicrobial (MIC = 22.5 ± 2.3‐48.3 ± 6.5 µg/mL)	[77]
Trichoderma hamatum	Bergenia purpurascens	China	**161**	Antimicrobial (51.0 ± 3.4%)	[78]
*Aspergillus puniceus*	*Eupatorium chinense*	China	**162** and **163**	PTP1B inhibitory (IC_50_ = 2.89 µM and 0.78 µM) and cytotoxic (IC_50_ = 1.45 µM and 1.28 µM)	[79]
Daldinia eschscholzii	Ceriops tagal	China	**164–166**	Antimicrobial (MIC = 6.25‐25 µg/mL)	[80]
Talaromyces pinophilus	Perilla frutescens	China	**167** and **168**	Cytotoxic (IC_50_ = 14.10 µM and 14.65 µM), anti‐osteoporotic (4 µM and 2 µM), and antimicrobial (MIC = 64 and 32 µg/mL)	[81]
Diaporthe pseudooculi	Sonneratia	China	**169**	Anti‐inflammatory (IC_50_ = 6.25 µM)	[82]
Arcopilus aureus	Ardisia mamillata	China	**170**	Antimicrobial (MIC = 6.25 µg/mL)	[83]
Alternaria sp.	Aconitum hemsleyanum	China	**171** and **172**	Antimicrobial (MIC = 16, 32, and 16 µg/mL)	[84]
*Fuscoporia* sp.	Mangrove	Malaysia	**173** and **174**	Neuroprotective (IC_50_ = 6.0 µM) and anti‐inflammatory (5.0 µM)	[85]
*Ascomycota* sp. and *Trichoderma harzianum*	*Ganoderma* sp.	China	**175** and **176**	Anti‐inflammatory (20 µM)	[86]
*Fusarium asiaticum*	*Artemisia argyi*	China	**177**‐**183**	Antimicrobial (MIC = 1 to 64 µg/mL)	[87]
Alternaria alstroemeriae	Catharanthus roseus	China	**184**	Antimicrobial (MIC = 3.125 and 1.5625 µg/mL)	[88]
*Talaromyces purpureogenus*	*Rhododendron molle*	China	**185**	XOD inhibitory (10 µM)	[89]
*Phomopsis* sp.	*Rhizophora mangle*	China	**186**–**188**	Antitumor (IC_50_ = 4.66 ± 0.35–21.69 ± 9.11 µM)	[90]
*Trichoderma protrudens*	*Hibiscus tiliaceus*	—	**189**	Cytotoxic (IC_50_ = 22.7 µM)	[91]
*Penicillium brefeldianum*	*Houttuynia cordata*	China	**190**	α‐glucosidase inhibitory (EC_50_ = 29.8 ± 9.4 µM)	[92]
*Fusarium* sp.	*Rhodiola rosea*	China	**191**‐**193**	Antimicrobial (MIC = 12.5–25 µg/mL	[93]
*Xylaria curta*	*Alpinia zerumbet*	China	**194**	Antimicrobial (MIC ‐ 6.3 µg/mL)	[94]
*Aspergillus aculeatus*	—	—	**195** and **196**	Herbicidal (IC_50_ = 3.01 ± 0.40 µM and 3.33 ± 0.52 µM)	[95]
*Aspergillus* sp.	*Kandelia candel*	China	**197**	Anti‐nonalcoholic steatohepatitis (potent)	[96]
*Aspergillus* sp.	*Kandelia candel*	China	**198**	Cytotoxic (IC_50_ = 28.4 µM)	[97]
Aspergillus sp.	Kandelia candel	China	**199**	Cytotoxic (IC_50_ = 6.81, and 15.40 µM)	[98]
Aspergillus terreus	Artemisia arborescens	—	**200**‐**204**	Cytotoxic (IC_50_ = 3.72–6.27 µM)	[99]
Aspergillus sp.	Heritiera littoralis	—	**205**	Insecticidal (93.96%)	[100]
Penicillium restrictum	Psammosilene tunicoides	China	**206** and **207**	Antimicrobial (MIC = 1 to 32 µg/mL)	[39]
Exserohilum rostratum	Kandelia candel	China	**208** ‐ **210**	α‐glucosidase inhibitory (IC_50_ = 0.093–0.250 mM)	[101]
*Talaromyces amestolkiae*	*Kandelia obovata*	China	**211**	Antiviral (EC_50_ = 2.50 ± 0.42 µM) and anti‐inflammatory (IC_50_ = 4.10 µM)	[102]
Thermothielavioides terrestris	Stemona mairei	China	**212**	Antimicrobial (10 mm)	[103]
Diaporthe arengae	Camellia oleifera	China	**213**	Antimicrobial (MIC = 16 µg/mL)	[104]
Alternaria malorum	Myoporum bontioides	China	**214** and **215**	Antimicrobial (MIC = 50 µg/mL)	[105]
*Protoventuria* sp.	*Vaccinium macrocarpon*	Canada	**216** and **217**	Antimicrobial (20 µL)	[106]
Aspergillus giganteus	Capparis carandas	Bangladesh	**218**	Antimicrobial (9 mm)	[107]
*Penicillium brocae*	*Curvularia spicifera*	—	**219**	Antimicrobial (MIC = 64 µg/mL)	[108]
*Talaromyces purpureogenus*	*Rhododendron molle*	China	**220**	XOD inhibitory (69.9% at 10 µM)	[89]
*Penicillium* sp.	—	—	**221**	Anti‐inflammatory (IC_50_ = 13.51 ± 6.70 µM)	[109]
Talaromyces funiculosus	Catharanthus roseus	—	**222**	Antimicrobial (MIC = 5.2–21.6 µg/mL) and cytotoxic (IC_50_ = 19.37 ± 0.1922.87 ± 0.23 µM)	[110]
Aspergillus sp.	—	China	**223** and **224**	Insecticidal (95.4% and 93.7%)	[111]
Bipolaris eleusines	Potatoes	China	**225** and **226**	Cytotoxic (IC_50_ = 14.48 and 17.99 µM)	[112]
*Chaetomium* sp.	*Vaccinium bracteatum*	China	**227** and **228**	Anti‐inflammatory (4 µM)	[113]
*Chaetomium virescens*	*Xylaria grammica*	China	**229**	Inhibitor and antitumor (potent)	[114]
*Penicillium brefeldianum*	*Houttuynia cordata*	China	**230‐233**	Cytotoxic (IC_50_ = 1.5 ± 0.1‐9.9 ± 0.9 µM)	[115]
Gliocladium sp.	Mahonia fortunei	China	**234**	Cytotoxic (IC_50_ = 9.05, and 19.25 µM)	[116]
Schizophyllum sp.	Bruguiera gymnorrhiza	China	**235**‐**239**	Anti‐inflammatory (10 µM)	[117]
Umbelopsis dimorpha	Vaccinium dunalianum	—	**240**‐**242**	Antimicrobial (MIC = 15.63–62.5 µg/mL)	[118]

## 2 Structural and Biological Activity Studies

### 2.1 Peptides

**FIGURE 5:**
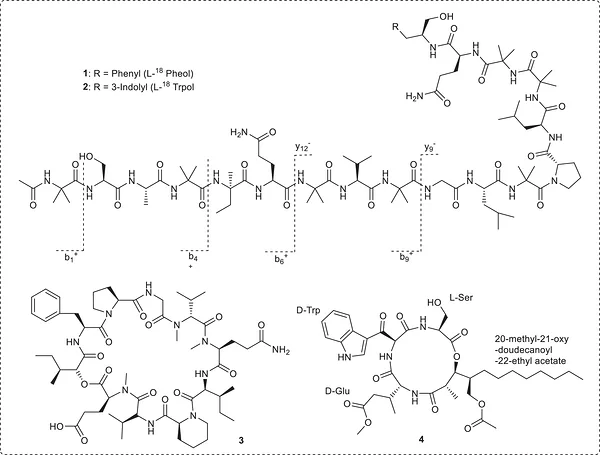
Chemical structures of compounds **1–4**.

Peptaibols are a distinctive group of linear fungal oligopeptides, typically consisting of 5 to 20 amino acid residues [24]. They are characterized by a high proportion of non‐proteinogenic amino acids, particularly α‐aminoisobutyric acid (Aib) and isovaline (Iva), along with an N‐terminal acetyl group, and a C‐terminal amino alcohol [25]. These unique structure features confer exceptional conformational stability and contribute to their diverse biological activities. Peptide metabolites have garnered considerable attention as promising therapeutic candidates due to their relatively low toxicity, high target selectivity, and ability to modulate various biological processes. Most fungal peptides have molecular weights ranging from 500 to 5000 Da, and many bioactive compounds have been isolated from endophytic fungi, especially species of *Trichoderma*, *Fusarium*, *Gliocladium*, and *Acremonium* [26, 27, 28, 29]. Reported biological activities include antioxidant [30], antimicrobial [31], antiviral [32], anti‐inflammatory [33], and anticancer effects [31, 34]. The broad biological activity of peptaibols is primarily attributed to their characteristic amphipathic α‐helical structures. The high abundance of Aib residues strongly promotes stable helical conformations that readily integrate into phospholipid bilayers. Upon membrane insertion, peptide helices self‐assemble into voltage‐dependent ion channels, causing membrane depolarization, uncontrolled ion flux, osmotic imbalance, and ultimately cell death [35]. This membrane‐disruptive mechanism fundamentally differs from that of most conventional antimicrobial agents, which typically target specific intracellular enzymes or metabolic pathways. Two previously undescribed peptaibols, trichorzin PA VI (1) and trichorzin PA II (**2**) (Figure 5), were isolated from *Trichoderma lentiforme* ML‐P8‐2, an endophyte obtained from healthy leaves of the mangrove plant *Bruguiera gymnorrhiza* [31]. Both compounds exhibited potent cytotoxic activity against MDA‐MB‐435, MDA‐MB‐231, HCT‐116, SNB‐19, PC‐3, and A549 cancer cell lines, with IC_50_ values ranging from 0.76 ± 0.68 to 27.18 ± 3.60 µM. They also demonstrated broad‐spectrum antimicrobial activity against all tested microorganisms, with MIC values of 1.56–6.25 µM, using cisplatin and ampicillin as positive controls [31]. Their pronounced biological activities are likely attributable to the abundance of Aib and Iva residues, which stabilize amphipathic α‐helices capable of efficiently penetrating phospholipid membranes and forming transmembrane pores. Consequently, the cytotoxic and antimicrobial activities of compounds **1** and **2** are primarily associated with membrane permeabilization rather than inhibition of specific intracellular molecular targets. In contrast to linear peptaibols, cyclic peptide metabolites generally possess macrocyclic depsipeptide frameworks that confer increased conformational rigidity, enhanced metabolic stability, and greater resistance to enzymatic degradation. A previously unreported cyclodepsipeptide (**3**, Figure 5) was isolated from the endophytic fungus *Phaeosphaeria* sp. XXH003, associated with *C. literatus* collected from the South China Sea [36]. Biological evaluation using a *Caenorhabditis elegans* life‐cycle model demonstrated antifibrotic activity against renal fibrosis at concentrations of 10‐20 µM, while additional assays revealed antiviral activity against vesicular stomatitis virus and herpes simplex virus at 15–20 µM [36]. Another new cyclic peptidearthrichitin derivative I (**4**) (Figure 5), was isolated from a co‐culture of endophytic *Aspergillus* sp. and *Penicillium* sp., associated with the mangrove plant *A. ilicifolius* L., collected in Guangxi, China [37]. Compound **4** exhibited selective cytotoxicity toward MHCC‐97H hepatocellular carcinoma cells with an IC_50_ value of 20.1 ± 0.9 µM, although only weak antimicrobial activity was observed against the tested bacterial strains [37]. The distinct biological properties of linear peptaibols and cyclic depsipeptides largely stem from their contrasting molecular architectures. Peptaibols primarily exert their biological effects through membrane permeabilization mediated by amphipathic α‐helices, whereas cyclic depsipeptides generally act via selective interactions with intracellular proteins, enzymes, or signaling pathways. Their pharmacological profiles are influenced by factors, such as macrocycle size, amino acid composition, stereochemistry, conformational flexibility, and the hydrophobicity of side chains. Together, these characteristics determine membrane permeability, target recognition, and binding affinity. These structural differences account for the broad‐spectrum antimicrobial activity typically associated with peptaibols and the comparatively greater target selectivity exhibited by cyclic peptide metabolites. Collectively, peptide metabolites produced by endophytic fungi share several conserved structural features, including the incorporation of non‐proteinogenic amino acids, N‐terminal acetylation, C‐terminal amino alcohols, and macrocyclic depsipeptide frameworks. These molecular characteristics enhance conformational stability, membrane affinity, resistance to proteolytic degradation, and biological selectivity. Among the peptide metabolites reported to date, trichorzin PA VI (**1**) and trichorzin PA II (**2**) stand out as particularly promising lead compounds due to their potent broad‐spectrum antimicrobial activity combined with low‐micromolar cytotoxic potency. Ecologically, these metabolites likely contribute to host defense by suppressing competing microorganisms and phytopathogens. Their well‐defined structural features, potent bioactivities, and emerging structure‐activity relationships highlight the considerable potential of fungal peptide metabolites as valuable scaffolds for developing next‐generation antimicrobial and anticancer agents.

### 2.2 Terpenoids and Steroids

Terpenoids constitute one of the largest and most structurally diverse groups of specialized metabolites synthesized by endophytic fungi and have garnered increasing interest due to their extensive pharmacological potential [14]. Recent studies have identified numerous previously unreported terpenoids belonging to the principal subclasses, including monoterpenes, sesquiterpenes, diterpenes, sesterterpenes, and triterpenes from fungi associated with plants, soil, and marine environments [38]. Representative terpenoid‐producing genera include *Trichoderma, Aspergillus, Eutypella*, and *Penicillium* [14, 39, 40]. Collectively, these metabolites exhibit diverse biological activities, such as anti‐inflammatory, antibacterial, antifungal, antimicroalgal, and cytotoxic effects, underscoring their value as promising scaffolds for drug discovery and pharmaceutical development [41].

#### 2.2.1 Meroterpenoids

**FIGURE 6:**
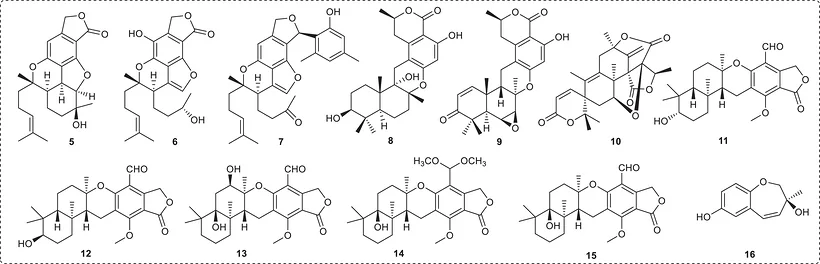
Chemical structures of compounds **5‐**‐**16**.

Three new meroterpenoids‐bisabosqual F (**5**) and stachybisbins J‐L (**6** and **7**) (Figure 6) were isolated from an undescribed endophytic fungus belonging to the family *Stachybotryaceae*, recovered from the roots of *Delphinium yunnanense* collected in Yunnan Province, China [42]. Cytotoxic evaluation against HL‐60, SMMC‐7721, A549, MCF‐7, and SW480 human cancer cell lines demonastrated pronounced antiproliferative activity with IC_50_ values ranging from 1.80 ± 0.08 to 17.76 ± 0.97 µM [42]. Their potent cytotoxicity may be attributed to the highly oxygenated meroterpenoid framework, which provides multiple interaction sites for molecular targets involved in cancer cell proliferation and survival. A novel meroterpenoid, peniclactone D (**8**, Figure 6), was isolated from the endophytic fungus *Penicillium* sp. GDGJ‐285, derived from the root of *Styrax tonkinensis* collected in Baise, Guangxi, China [43]. In lipopolysaccharide (LPS)‐stimulated RAW 264.7 macrophage, compound **8** significantly exhibited nitric oxide (NO) production, with an IC_50_ value of 18.50 ± 1.26 µM, demonstrating greater potency than positive control dexamethasone (IC_50_ = 33.61 ± 4.45 µM) [43]. This enhanced activity may be attributed to oxygenated functional groups that facilitate the modulation of inflammatory mediators, although further mechanistic studies are required to confirm this. The fungal strain *T. primulinus* H21, isolated from *E. sikkimensis* collected in Yunnan, China, yielded a new meroterpenoid compound (**9**, Figure 6) that exhibited anti‐inflammatory activity in lipopolysaccharide (LPS)‐stimulated RAW 264.7 macrophage [44]. Compound **9** showed the strongest inhibition of nitric oxide (NO) production, with an IC_50_ value of 27.19 µM. No significant cytotoxicity was observed in the MTT assay at the tested concentrations, supporting a genuine anti‐inflammatory effect [44]. Another previously undescribed meroterpenoid, talaromeroterpenoid B (**10**) (Figure 6), was isolated from the mangrove‐derived fungal strain *Talaromyces* sp. JNQQJ‐4, collected in Anhui, China [45]. Compound **10** significantly suppressed NO production in lipopolysaccharide (LPS)‐stimulated RAW 264.7 macrophages, exhibiting an IC_50_ value of 8.25 µM, wich is lower than that of the positive control (IC_50_ = 11.33 µM) [45]. Fermentation of the endophytic fungus *Diaporthe* sp. XC1211, isolated from the root tissues of *R. simsii* Planch., collected in Hubei, China, yielded five new meroterpenoids (**11‐**‐**15**) (Figure 6) [46]. Their immunomodulatory properties were evaluated using murine splenic T and B lymphocytes. Compound **12** showed pronounced immunosuppressive activity by inhibiting both ConA‐induced T‐cell proliferation (IC_50_ = 11 µM) and LPS‐induced B‐cell proliferation (IC_50_ = 6.7 µM). Compounds **13** and **15** selectively inhibited B‐cell proliferation, with IC_50_ values of 8.5 and 3.8 µM, respectively, whereas compound **11** displayed cytotoxicity against HeLa cells (IC_50_ = 9.6 ± 1.6 µM) [46]. A new isoprenyl‐phenol‐type meroterpenoid, asperphenol A (**16**, Figure 6), was isolated from the endophatic fungus *Aspergillus* sp. GXNU‐Y65, which is associated with the mangrove plant *K. candel* collected in Beihai, China [47]. Cytotoxic evaluation against A549 and T24 human cancer cell lines revealed selective activity toward T24 cells, with an IC_50_ value of 26.71 µM [47].

#### 2.2.2 Sesquiterpenoids

**FIGURE 7:**
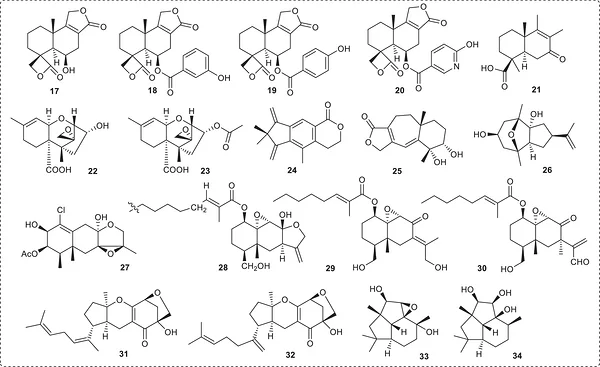
Structures of compounds **17‐**‐**34**.

Five previously undescribed drimane‐type sesquiterpenes, spinulactones A‐F (**17‐**‐**21**) (Figure 7), were isolated from the cultured mycelia of the fungal strain *P. spinulosum* 9‐1, an endophytic fungus obtained from the roots of *E. henryi* Oliv., collected in Hubei Province, China [48]. The isolated metabolits were evaluated for in vitro antifungal, antiaging, and proangiogenic activities. Compounds (**17‐**‐**21**) exhibited broad‐spectrum antifungal activity against *A. alternata, F. graminearum, F. oxysporum*, and *Botrytis cinerea*, with MIC values ranging from 12.5 to 50 µg/mL. Additionally, compound **21** alleviated D‐galactose‐induced cellular senescence in GES‐1 gastric epithelial cells, indicating promising antiaging potential [48]. Two previously undescribed trichothecene‐derivated sesquiterpens, compounds **22** and **23** (Figure 7) were isolated from the endophytic fungus *F. equiseti* Z8, which is associated with the leaves of *C. roseus* (L.) [49]. Both metabolites exhibited significant antiproliferative activity against the non‐small‐cell lung cancer cell lines A549 and NCI‐H1944, with IC_50_ values ranging from 11.05 ± 0.28 to 24.87 ± 0.21 µM. Although their activity was lower than that of the positive control, adriamycin (IC_50_ = 0.22–0.81 µM), these results demonstrate that trichothecene derivatives remain promising anticancer scaffolds [49]. Fermentation of the endophytic fungus *C. nigricolor* F5, isolated from the medicinal plant *M. fortunei*, yielded a previously undescribed sesquiterpene featuring a rare 9,10‐seco‐15‐nor‐isoilludalane carbon skeleton (**24**, Figure 7) [50]. Compound **24** demonstrated significant neuroprotective and anti‐neuroinflammatory effects in LPS‐stimulated BV2 microlial cells at 10 µg/mL by suppressingp65 phosphorylation and preventing its nuclear translocation, thereby inhibiting activation of the TLR4/NF‐κB signaling pathway [50]. A novel sesquiterpene, guxinusocapsa A (**25**,Figure 7), was isolated from the fermentation broth of the endophytic fungus Aspergillus sp. GXNU R1, which is associated with the mangrove plant Kandelia candel collected in Guangxi, China [51]. Compound **25** exhibited potent insecticidal activities against the Asian citrus psyllid (Diaphorina citri), causing 95% mortality at 1000 mg/kg [51]. The marine algal endophyte *P. oxalicum* 2021CdF‐3, isolated from *R. confervoides*, produced a previously undescribed sesquiterpene, penicivetivane (**26**) (Figure 7) [52]. Antimicrobial evalution revealed significant activity against *E. coli* and *S. aureus* with MIC values of 4 and 2 µg/mL, respectively, while weaker activity was observed against [52]. The endophytic fungus *A. cycadis*, isolated from *S. acutum*, yielded a new eremophilane‐type sesquiterpenoid, acroeremophilane C (**27**, Figure 7) [53]. Compound **27** exhibited potent cytotoxicity against the human cancer cell lines HeLa, A549, and SMMC‐7721, with IC_50_ values of 2.89 ± 0.54, 4.55 ± 0.80, and 5.71 ± 1.22 µM, respectively [53]. The endophytic fungus *Diaporthe* sp. BCC69512 isolated from the leaves of *E. littoralis* collected in Trang Province, Thailand, produced three rare eremophilane derivatives: diaportheremopholins B, E, and F (**28**‐**30**) (Figure 7) [54]. Biological evaluation demonstrated that all three compounds exhibited antitubercular activity, with MIC values ranging from 25 to 50 µg/mL. Additionally, they inhibited the growth of *B. cereus*, showing antibacterial activity within the same MIC range [54]. Two previously undescribed sesquiterpene‐shikimate hybrids, compouds **31** and **32** (Figure 7) were isolated from the endophytic fungus *P. capitalensis*, which is associated with *L. chinense* var. rubrum, collected in Jiangsu, China [55]. Their anti‐inflammatory activity was evaluated by measuring the inhibition LPS‐induced nitric oxide production in BV2 microglial cells. Compounds **31** and **32** inhibited nitric oxide production with IC_50_ values of 21.65 ± 1.33 µM and 12.19 ± 1.08 µM, respectively, indicating promising anti‐inflammatory potential [55]. The endophytic fungus *C. camelliae* LP76, associated with *C. taliensis*, produced two new sesquiterpenes, collcameins A (**33**) and B (**34**) (Figure 7) [56]. Both compounds exhibited antifungal activity against several phytopathogenic fungi, including *F. oxysporum, C. versicolor, A. brassicicola, F. solani*, *B. cinerea, G. trabeum*, and *F. graminearum*. The strongest activity was observed against *A. brassicicola*, with MIC values of 31.25 µg/mL for both metabolites [56].

#### 2.2.3 Diterpenoids

**FIGURE 8:**
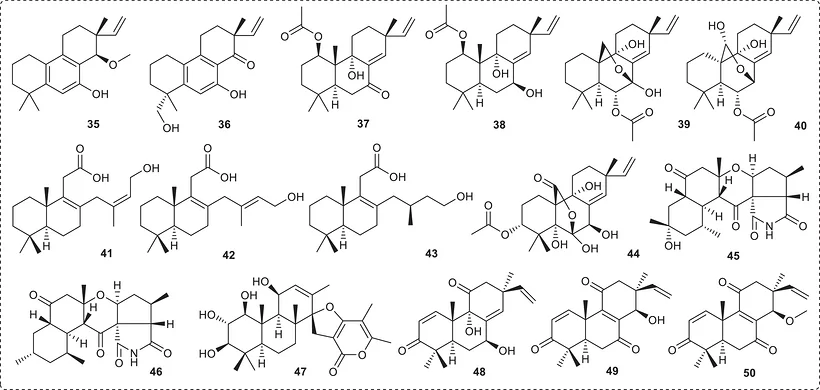
Structures of compounds **35–50**.

Diterpenoids produced by endophytic fungi exhibit significant structural diversity and have garnered increasing attention due to their anti‐inflammatory, immunomodulatory, and cytotoxic properties. Recent studies have identified several novel diterpenoid scaffolds, including isopimarane, labdane, pimarane, and spiroditerpenoid‐type metabolites, all of which show promising pharmacological potential. Six new isopimarane‐type diterpenoids, pleosmaranes A‐F (**35**‐**40**) (Figure 8), were isolated from the mangrove‐derived endophytic fungus *Pleosporales* sp. HNQQJ‐1, obtained from the leaves of *K. candel* collected in Hainan, China [57]. Evolution of their anti‐inflammatory activity in LPS‐stimulated RAW 264.7 macrophages revealed that compounds **36** and **38** effectively inhibited nitric oxide production, with IC_50_ values of 19 and 25 µM, respectively [57]. The endophytic fungus*Talaromyces* sp. JNQQJ‐4 isolated from healthy leaves of Kandelia obovata collected in Guangzhou, China, yielded four diterpenoids featuring a 1,2,3,4,4a,5,6,8a‐octalin skeleton, named talaroacids A‐D (**41**‐**43**) (Figure 8), along with the isopimarane diterpenoid, talaromarane A (**44**, Figure 8) [58]. Notably, talaromarane A contains a rare 2‐oxabicyclo [3.2.1] octane moiety embedded within the isopimarane diterpenoid framework. Biological evaluation demonstrated that compounds **41‐**‐**44** exhibited significant anti‐inflammatory activity in RAW 264.7 macrophages, with IC_50_ values ranging from 4.59 to 21.60 µM [58]. Feng et al. [59] reported two novel labdane‐type diterpenoids, diaporlabanoids A (**45)** and B (**46**) (Figure 8), isolated from the fermentation broth of the endophytic fungus *D.phaseolorum* H3‐2, associated with the mangrove plant *C. tagal* collected in Hainan, China. Their immunosuppressive activities were evaluted using concanavalin A (ConA)‐stimulated murine splenocyte proliferation assays. Compound **46** exhibited the strongest inhibition of ConA‐induced T‐cell proliferation, compound **45** demonstrated a more favorable safety profile by maintaining higher viability of normal splenocytes (IC_50_ value of 10.831 ± 2.54 µM). In contrast, compound **46** showed greater cytotoxicity (IC_50_ = 13.13 ± 2.03 µM). Importantly, compound **45** displayed substantially lower cytotoxicity than the clinically used immunosuppressant *cyclosporin* A, suggesting an improved therapeutic safety margin [59]. A previously undescribed polycyclic spiroditerpenoid compound **47** (Figure 8) was isolated from the endophytic fungus *P. bialowiezense* ZBWPQ‐27, which is associated with the medicinal plant *E. neriifolia*, collected in Yunnan, China [60]. Compound **47** exhibited significant immunosuppressive activity by inhibiting both ConA‐induced T‐cell proliferation and LPS‐induced B‐cell proliferation, with IC_50_ values of 7.3 ± 0.8 and 6.3 ± 1.0 µM, respectively [60]. The endophytic fungus *Pseudoplectania* sp. EL000327 produced three previously undescribed pimarane‐type diterpenoids, named plectalibertellenones A‐C (**48**‐**50**) (Figure 8) [61]. Cytotoxicity evaluation demonstrated that all three metabolites inhibited the proliferation of Caco‐2 and HaCaT cell lines, with IC_50_ values ranging from 17.4 to 80.7 µM [61].

#### 2.2.4 Triterpenoid and Steroids

**FIGURE 9:**
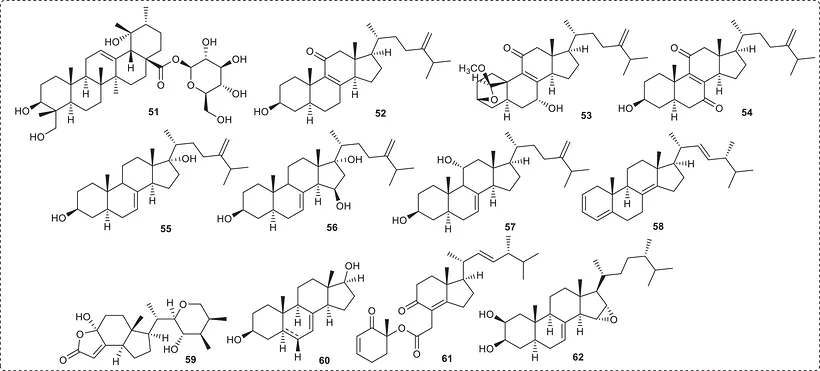
Structures of compounds **51**–**62**.

Triterpenoids and steroids produced by endophytic fungi represent an important class of terpenoid metabolites characterized by diverse oxidative modifications and a wide range of biological activities. Recent studies have identified numerous previously unreported fungal triterpenoids and steroids exhibiting promising antimicrobial, anti‐inflammatory, and cytotoxic properties. The endophytic fungus *P. macrosclerotiorum*, isolated from *I. pubescens* Hook. et Arn., produced a previously undescribed triterpenoid, pedunculoside (**51)** (Figure 9) [62]. Compound **51** demonstrated antifungal activity against *C. albicans*, with MIC value of 0.78 mg/mL [62]. Seven new steroidal metabolites, camphoratins K‐O (**52**‐**57**) (Figure 9), were isolated from the endophytic fungus *Aspergillus* sp. TZS‐Y4, which is associated with the leaves of *P. heterophylla* (Miq.) Pax, collected in Liaoning Province, China [63]. Their anti‐inflammatory activity was evaluated in LPS‐stimulated RAW 264.7 macrophages, where all compounds inhibited nitric oxide production at 10 µM. Among them, compound **56** exhibited the strongest anti‐inflammatory activity and were identified as the most promising lead for further pharmacological investigation [63]. A previously undescribed ergostane‐type steroid, aspergteroid (**58)**, (Figure 9), was isolated from the endophytic fungus *Aspergillus* sp. YUD20004, which is associated with the tubers of *Z. officinale* Roscoe collected in Yunnan, China [64]. Cytotoxic evaluation against human cancer cell lines SMMC‐7721, HL‐60, A549, SW480, and MDA‐MB‐231 demonstrated exceptionally potent activity against SMMC‐7721 hepatocellular carcinoma cells, with an IC_50_ value of 0.40 µM. This represents one of the most active steroidal metabolites reported from endophytic fungi [64]. The endophytic fungus *Trichoderma* sp. GLQ02, isolated from *G. lucidum* collected in Guangdong, China, produced an uncommon nor‐steroid (**59)**, (Figure 9) [65]. In lipopolysaccharide (LPS)‐stimulated RAW 264.7 macrophages, compound **59** inhibited a nitric oxide producion by 41.17% at 20 µM indicating moderate anti‐inflammatory activity [65]. Chemical investigation of the endophytic fungus *P. fellutanum*, isolated from *E. helioscopia* L. collected in Shaanxi, China. led to the identification of a new steroid, acrocalysterol F (**60)**, (Figure 9) [66]. Compound **60** exhibited cytotoxic activity against RKO, and HCC‐1806 human cancer cell lines, with IC_50_ values of 22.56 ± 2.30 and 18.15 ± 1.05 µM, respectively. It also demonstrated antifungal activity against several phytopathogenic fungi, including *F. culmorum, Valsa mali Miyabe*, and *F. solani* [66]. A novel steroid, bipolaristeroid A (**61)** (Figure 9), was isolated from the endophytic fungus *B. maydis* [67]. Biological evaluation demonstrated broad‐spectrum cytotoxicity against the human cancer cell lines HepG2, A549, SW620, and C4‐2B, with IC_50_ values of 7.94 ± 0.02, 5.11 ± 0.09, 5.13 ± 0.12, and 3.83 ± 0.16 µM, respectively indicating promising anticancer potential [67].Another previously undescribed steroid, acrocalysterol D (**62)** (Figure 9), was isolated from the endophytic fungus *A. cycadis*, which is associated with *S. acutum* (Thunb.) Rehd. et Wils., collected in Shaanxi, China [68]. Cytotoxic evaluation against HeLa and RKO cancer cell lines revealed IC_50_ values of 27.02 ± 3.19 and 22.13 ± 0.17 µM, respectively, using etoposide as the positive control [68]. Terpenoids represent one of the most structurally diverse groups of secondary metabolites produced by endophytic fungi, including meroterpenoids, sesquiterpenoids, diterpenoids, triterpenoids, and steroids. Their biological properties are closely linked to structural features, such as oxidation patterns, stereochemical configurations, ring rearrangements, and hybridization with aromatic or polyketide‐derived moieties. Generally, highly oxygenated terpenoids exhibit enhanced anti‐inflammatory activity, whereas rigid diterpenoid and steroidal frameworks are more commonly associated with pronounced cytotoxic and immunomodulatory effects. Meroterpenoids, due to their hybrid polyketide‐terpenoid architecture, often display broad‐spectrum pharmacological activities. Among the compounds reviewed, aspergteroid (**58**) demonstrated the highest cytotoxic potency (IC_50_ = 0.40 µM), while talaromarane A (**44**) is notable for its unprecedented 2‐oxabicyclo[3.2.1]octane diterpenoid skeleton. Steroidal metabolites possess a conserved tetracyclic core that undergoes extensive structural diversification through hydroxylation, oxidation, side‐chain modification, and occasional ring contraction. Their biological activity is largely influenced by the number and position of hydroxyl and carbonyl groups, double bonds, and side‐chain substitutions. Oxygenated ergostane derivatives generally exhibit superior cytotoxic activity, whereas polyhydroxylated steroids are more frequently linked to anti‐inflammatory effects. Notably, aspergteroid (**58**) displayed submicromolar cytotoxicity, and bipolaristeroid A (**61**) exhibited broad‐spectrum anticancer activity. Collectively, the remarkable structural diversity and well‐defined structure–activity relationships of fungal terpenoids and steroids underscore their significance as promising scaffolds for developing anticancer, anti‐inflammatory, antimicrobial, and immunomodulatory therapeutics.

### 2.3 Alkaloids and Nitrogen Compounds

**FIGURE 10:**
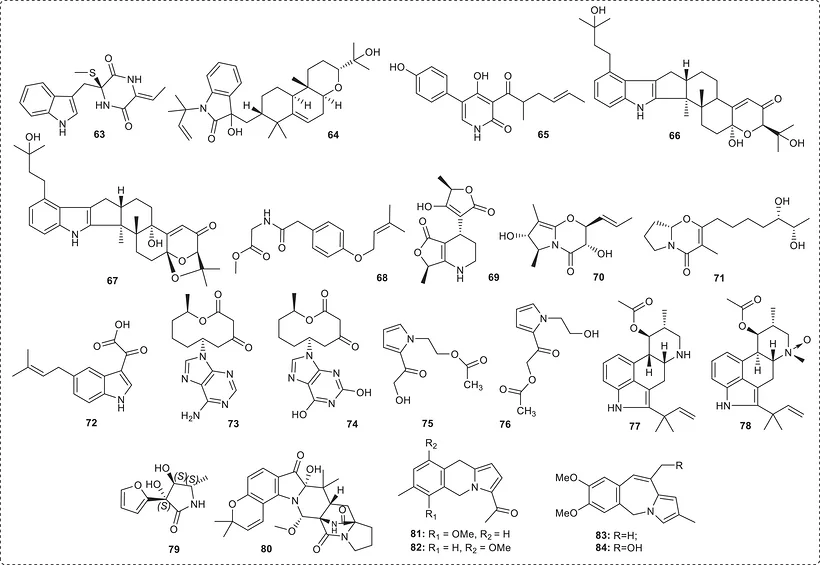
Structures of compounds **63**–**84**.

Alkaloids and other nitrogen‐containing specialized metabolites form an important group of natural products because of their structural variety and the broad spectrum of biological activities they display, which them promising options for both pharmaceutical and agricultural uses [10, 69]. Endophytic microorganisms show remarkable metabolic flexibility, and because they live in close association with their host plants, they can produce structurally diverse nitrogenous metabolites. These metabolits may imitate, support, or even outperform the biological effects of plant derived alkaloids [70]. Such compounds cover a wide range of alkaloid frameworks, including indole, quinoline, isoquinolone, pyridine, pyrrolidine, and quinazoline derivatives. In addition to the well‐known alkaloids, endophytes also generate many other nitrogen‐containing substances, such as peptides, diketopiperazines, amides, and various nitrogenous heterocycles. Many of these have antimicrobial, anticancer, antioxidant, and enzyme‐inhibitory effects [71]. Key alkaloid‐producing endophytes include fungal genera such as Aspergillus, Penicillium, Fusarium, Claviceps, Colletotrichum, and Alternaria, along with bacterial genera such as Streptomyces, Bacillus, and Pseudomonas [72, 73]. Their biosynthesis is largely directed by specialized biosynthetic gene clusters, particularly non‐ribosomal peptide synthetase (NRPS) and hybrid polyketide synthase–NRPS (PKS–NRPS) pathways. Together, these systems help drive the exceptional structural diversity seen in nitrogenous metabolites produced by endophytes [74]. The endophytic fungus *B. ochroleuca* SLJB‐2, isolated from the stems of *S. doederleinii* collected on Diaoluo Mountain in Hainan Province, China, produced a previously undescribed indole‐derived diketopiperazine, named ochrolines B (**63**) (Figure 10) [75]. Compound **63** showed antibacterial activity against *S. aureus*, methicillin‐resistant *S. aureus* (MRSA), and *E. coli*, with MIC value of 64 µg/mL. It also exhibited cytotoxic effects againstthe MCF‐7 and HeLa cell lines, with IC_50_ values of 20.27 and 25.84 µM, respectively [75]. A new indole diterpenoid, asperdole D (**64**, Figure 10), was isolated from *Aspergillus* sp. TJ403‐YJ22, an endophytic fungus found in association with the lichen *Umbilicaria* sp. from Nanquan Mountain in Guizhou Province China [76]. The compound's interaction with the molecular target was verified using cellular thermal shift assay (CETSA) and bio‐layer interferometry (BLI). Compound **64** functioned as a selective agonist of the farnesoid X receptor (FXR), showing an EC_50_ value of 4.1 ± 0.3 µM, which underscores its promise as a lead candidate for treating cholestatic disorders [76]. Tang et al. [77] also described a previously unreported pyridinone derivative, (±)‐porripyridone (**65**, Figure 10), which was isolated from the fermentation broth of the endophytic fungus *A. porri* #25. This fungus had been obtained from the mangrove plant *K. candel* (Linn.) Druce collected in Guangzhou, China. Compound **65** showed moderate cytotoxicity against human prostate cancer cell line VCaP with an IC_50_ value of 22.94 ± 0.53 µM [77]. In addition, two previously unreported indole alkaloids, brefeldindoles E and F (**66** and **67**, Figure 10), were isolated from the ethyl acetate extract of *P. brefeldianum* GXIMD 02511. This strain was collected from mangrove rhizosphere sediment obtained from Weizhou Island, China [78]. Both metabolites inhibited receptor activator nuclear factor‐κB ligand (RANKL)‐induced osteoclast differentiation at 20 µM, with no detectable cytotoxicity toward bone marrow‐derived macrophages. Mechanistic investigations revealed that compound **66** suppressed NF‐κB signaling by blocking the nuclear translocation of NFATc1 and by lowering the expression of the osteoclast fusion marker DC‐STAMP [78]. Preliminary structure–activity relationship analysis further indicated that the Δ^13 double bond in compound **66** and the added 1,3‐dioxolane ring in compound **67**, are important structural elements likely responsible for their biological activities [78]. An endophytic fungus, *F. oxysporum* LZC03, was isolated from the roots of *Salvia officinalis* collected in Anhui, China. From this source, the fungus produced a nitrogen‐containing metabolite that had not been reported before: methyl 2‐(4‐((3‐methylbut‐2‐en‐1‐yl)oxy)phenyl)acetylglycinate (**68**, Figure 10) [79]. Compound **68** showed cytotoxic effects against the HCT‐116, ASPC‐1, and PC‐3 cell lines, with IC_50_ values of 10.15, 12.56, and 25.34 µM, respectively.It also exhibited antioxidant properties, with IC_50_ values of 15.43 ± 1.00 and 30.23 ± 7.45 µg/mL in the DPPH and ABTS radical scavenging assays, respectively [79]. A previously undescribed alkaloid, penifuranone A (**69**, Figure 10), was isolated from *P. crustosum* SCNU‐F0006, an endophytic fungus obtained from the mangrove plant *A. ilicifolius* collected in Guangdong, China [80]. Compound **69** displayed antifungal activity against *F. oxysporum* and *P. italicum* (MIC of 0.25 mg/mL), and antibacterial activity against *P. aeruginosa*, (MIC of 0.5 mg/mL) [80]. The endophytic fungus *P. sizovae*, isolated from fresh tissues of *F. sinkiangensis* collected in Xinjiang, China, produced two previously unreported alkaloids: ferupencines A‐E (**70** and **71**, Figure 10) [81]. Both compounds showed moderate cytotoxicity against HeLa, SGC‐7901, and SHG‐44 cancer cell lines, with IC_50_ values spanning from 14.2 ± 1.5 to 23.8 ± 0.7 µM [81]. A novel indole derivative, 5‐(3′,3′‐dimethylallyl)indole‐3‐oxoacetic acid (**72**, Figure 10), was isolated from the endophytic fungus *C. globosum* BCC71876, This fungus was isolated from twigs of *A. crassna* collected in Phetchabun Province, Thailand [82]. In biological testing, the compound showed moderate antimalarial activity (IC_50_ value of 13.04 µM) as well as cytotoxicity toward Vero cells, with an (IC_50_ = 6.96 µM), whereas only weak antimicrobial activity was detected [82]. Two previously undescribed secondary metabolites, penicirestricols C (**73**) and D (**74**) (Figure 10), were isolated from the endophytic fungus *P. restrictum*, which is associated with the roots of *P. tunicoides* collected in Yunnan, China [83]. Both compounds demonstrated antifungal activity against *A. tenuissima* and *F. asiaticum*, with MIC values ranging from 1 to 32 µg/mL [83]. Two new N‐alkylated pyrrole derivatives (**75** and **76**, Figure 10) were isolated from the endophytic fungus *P. oxalicum*, associated with *Cedrus* spp. collected in Nantong, China [84]. Both metabolites exhibited wide‐ranging cytotoxic activity against A549, SK‐OV‐3, HeLa, XF498, and HCT‐115 cell lines, with IC_50_ values ranging from 7.86 ± 0.81 to 29.65 ± 2.33 µM. The most potent activity was observed against A549 cells, with IC_50_ values of 7.86 ± 0.81 µM for compound **75** and 9.37 ± 0.42 µM for compound **76** [84]. Two previously undescribed ergot alkaloids, fumigaclavines K and M (**77** and **78**, Figure 10), were isolated from the endophytic fungus *Aspergillus* sp. DVXT‐221, obtained from the mangrove plant *R. stylosa* collected in Nam Dinh, Vietnam [85]. Both metabolites suppressed inflammatory responses in LPS‐stimulated RAW 264.7 macrophages, with IC_50_ values of 5.3 ± 0.2 µM and 20.5 ± 0.8 µM respectively [85]. A new 2‐pyrrolidinone derivative, cladosporlactam (**79**, Figure 10), was obtained from the endophytic fungus *Cladosporium* sp., which lives in association with *R. tephrodes* Hance [86]. Compound **79** showed targeted antimicrobial effects against *S. aureus* and *B. subtilis*, with MIC values of 8 and 16 µg/mL, respectively, but it was much less effective against the other microorganisms that were tested [86]. A novel prenylated indole alkaloid aspertaichamide A (**80**, Figure 10), containing a bicyclo[2.2.2]diazaoctane skeleton, was isolated from *A. taichungensis* 299, an endophyte associated with the marine red alga *G. amansii* collected in Yantai, China [87]. Compound **80** displayed cytotoxic activity against multiple human cancer cell lines, with the strongest effect on AGS gastric cancer cells (IC_50_ = 1.7 ± 0.1 µM), then on A549 (19.2 ± 0.9 µM) and HeLa (23.4 ± 0.6 µM) cells [87]. Two previously unreported pyrroloisoquinoline metabolites, cigarpyrro A and B (**81** and **82**, Figure 10), were isolated from the endophytic fungus *A. puniceus*, which is associated with cigar tobacco gathered in Yunnan, China [88]. Both compounds showed broad‐spectrum antimicrobial activity against *S. aureus, E. coli, S. pyogenes, B. subtilis*, and *Proteus* spp., generating inhibition zones of 15.4 ± 1.8 to 25.2 ± 1.6 mm [88]. Both compounds exhibited potent antimicrobial activity against all tested pathogenic microorganisms, with inhibition zone diameters ranging from 15.4 ± 1.8 to 25.2 ± 1.6 mm [88]. The endophytic fungus *Aspergillus* fumigatus YNCA23‐26, isolated from cigar tobacco collected in Jiangchuan County, China, produced two previously undescribed benzoazepine derivatives (**83** and **84**) (Figure 10) [89]. Both metabolites exhibited antibacterial activity against several Nitrosomonas species, including *N. communis, N. nitrosa, N. multiformis, N. winogradskyi*, and *N. inopinata*. with inhibition zone diameters ranging from 14.0 ± 1.8 to 22.4 ± 2.2 mm [89].

**FIGURE 11:**
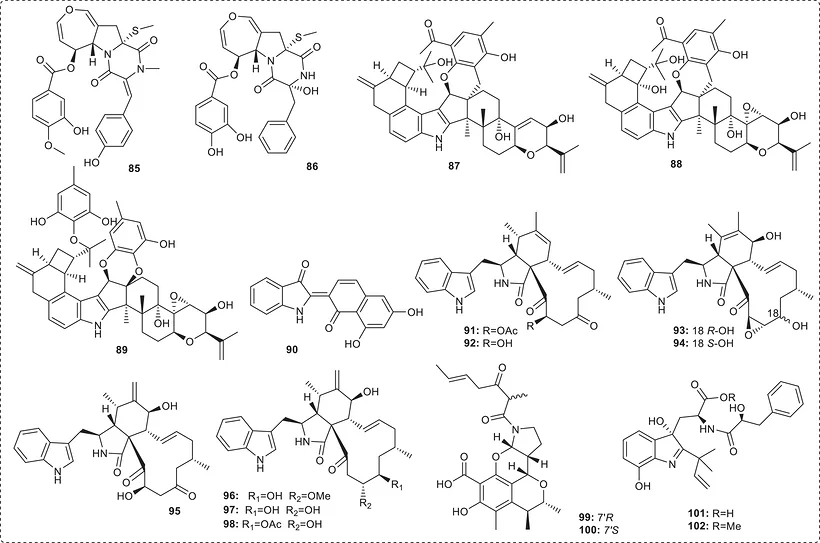
Structures of compounds **85**–**102**.

A pair of previously metabolites that had not been described before, asperstrins F and G (**85** and **86**, Figure 11), were isolated from the endophytic fungus *A. nidulans*. This fungus was found in association with the annelid *W. pigra* Whitman collected in Hubei Province, China [90]. When evaluated biologically, both compounds selectively inhibited butyrylcholinesterase (BuChE), with IC_50_ values of 9.49 ± 0.51 µM and 2.03 ± 0.55 µM, respectively. In contrast, only weak effects were seen on acetylcholinesterase (AChE) and the tested human cancer cell lines (HL‐60, A549, SMMC‐7721, MCF‐7, and SW480) [90]. Three previously unreported indole diterpenoids monoclavatol‐secopenitrems A and B (**87** and **88**) (Figure 11), and bis‐5‐methylpyrogallol‐secopenitrem (**89)** (Figure 11) were isolated from the endophytic fungus *P. bulbillosa* KNI755. This fungus was obtained from the medicinal plant *P. lingua* collected in Hubei Province, China [91]. Compounds **87‐**‐**89** significantly inhibited T‐cell proliferation triggered by Concanavalin A (ConA) with EC_50_ values of 13.0, 4.6, and 13.0 µM, respectively. They also suppressed lipopolysaccharide (LPS)‐induced B‐cell proliferation for compounds **87**–**88,** showing EC_50_ values of 7.9 and 7.6 µM, respectively. Overall, these results point to promising immunosuppressive activity [91]. Li et al. [92] isolated a previously undescribed indigotin alkaloid, talarindigotin A (**90**, Figure 11) from the mangrove‐derived endophytic fungus Talaromyces amestolkiae SCNU‐F0041. This fungus is associated with the leaves of Kandelia obovata collected in Guangdong Province, China. Compound **90** showed strong cytotoxic effects on HepG2, Huh7, HeLa, and HCT116 cell lines, with IC_50_ values of 2.74 ± 0.20, 2.08 ± 0.25, 4.58 ± 0.18, and 3.33 ± 0.43 µM, respectively [92]. Duan et al. [93] reported ten previously undescribed metabolites, chaepseubakerins A‐J (**91**–**98**) (Figure 11), isolated from the endophytic fungus *P. bakeri* P1‐1‐1. This fungus was collected from the roots of *M. tenue* subsp. sullivantii Vitt. (*Orthotrichaceae*), in Hubei Province, China. All of the compounds displayed cytotoxic activity against a range of human cancer cell lines, including A549, A427, HCT‐116, HT‐29, HeLa, HepG2, MCF‐7, and LO2, with IC_50_ values spanning from 2.9 ± 0.3 to 27.8 ± 1.2 µM. Mechanistic studies also showed that compound **91** triggered apoptosis and produced dose‐dependent G_2_/M phase cell cycle arrest in A549, HeLa, and HCT‐116 cells [93]. Two previously undescribed citrinin‐derived metabolites (**99** and **100**, Figure 11) were isolated from the endophytic fungus *P. citrinum* GZWMJZ‐836, which was associated with the leaves of *D. roosii* Nakaike, collected in Guizhou Province, China [94]. Both compounds showed antibacterial activity against *C. perfringens*, with MIC values of 62.5 and 31.3 µM, respectively. In addition, compound **100** inhibited *S. aureus* and *R. solanacearum*, (MIC 62.5 µM), while compound **99** exhibited antifungal activity against *S. sclerotiorum*, with an IC_50_ value of 61.9 ± 1.8 µM [94]. The endophytic fungus *A. alabamensis* SYSU‐6778, isolated from *E. acoroides* collected on Hainan Island, China, produced two previously undescribed diketomorpholine alkaloids (**101** and **102**, Figure 11) [95]. Both metabolites inhibited the fish pathogen *E. ictaluri*, with MIC values of 10.0 µg/mL. Activity against other microorganisms tested was relatively weak, suggesting selective antibacterial potential [95].

#### 2.3.1 Cytochalasins Alkaloids

**FIGURE 12:**
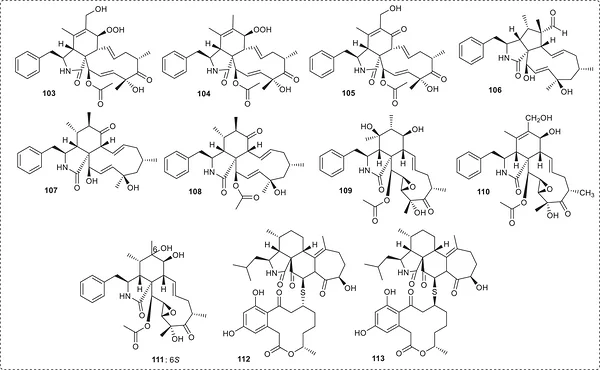
Structures of compounds **103‐**‐**113**.

Cytochalans are a distinctive group of fungal alkaloids defined by a highly modified perhydroisoindolone core fused to a macrocyclic ring system. Their biological effects mainly come from disturbing the behavior of actin filaments. Specifically they bind to the barbed ends of filamentous actin, polymers and disrupting actin polymerization dynamics. Changes made to the macrocyclic structure, such as hydroxylation, epoxidation, oxidation and the addition of extra heterocyclic or sulfur‐containing groups—play a major role in how these compounds interact with actin, and they ultimately affect their cytotoxic, antimicrobial, and other pharmacological activities.Three cytochalasans that had not been reported before xylariachalasins A‐C (**103**‐**105**) (Figure 12), were isolated from the endophytic fungus *X. arbuscula* QYF. This fungus was found living in the leaves of the mangrove plant *K. candel* collected in Hainan, China [96]. When tested biologically, the three compounds showed selective antimicrobial effects. Compound **103** inhibited *C. albicans* with an MIC value of 25 µM. Metabolite **104** showed antibacterial activity against *S. aureus* and methicillin‐resistant *S. aureus* (MRSA), with MIC values of 25 µM and 12.5 µM, respectively. Compound **105** was active against *P. aeruginosa*, with an MIC value of 25 µM [96]. Next, three more cytochalasan derivatives diacytochalasin A (**106**) and cytochalasins SY2 and SY3 (**107**‐**108**) (Figure 12) were isolated from the endophytic fungus *D. hongkongensis*, which is associated with *Strychnos nux‐vomica* [97]. In cytotoxicity tests using HGC27, HCT‐116, U87, MCF‐7, and HepG2 cell lines, compounds **106** and **107** were the most effective against HGC27 gastric cancer cells, with IC_50_ values of 1.35 and 3.59 µM, respectively. Meanwhile compound **108** showed its strongest activity against HepG2 cells, with an IC_50_ value of 9.93 µM [97].Similarly, the endophytic fungus *Rosellinia* sp. Glinf021, isolated from the medicinal plant *G. inflata* collected in Xinjiang, China, produced three new cytochalasans: rosellichalasins (**109**–**111**) (Figure 12) [98]. Among these compounds, **109** showed selective cytotoxicity against BGC823 gastric cancer cells (IC_50_ = 21.8 µM). Compound **111** showed only moderate activity against MDA‐MB‐231 breast cancer cells, with an IC_50_ of 21.4 µM [98]. Finally, structurally distinctive pair of sulfur‐containing heterodimeric cytochalasans, sucurchalasins A and B (**112** and**113**, Figure 12), was isolated from the endophytic fungus *Aspergillus spelaeus* GDGJ‐286. The fungus was obtained from fresh tissues of *Sophora tonkinensis* collected in Guangxi, China [99]. Unlike typical cytochalasans, these metabolites contain an unusual sulfur‐bridged connection that links a cytochalasan framework to a macrolide unit. This creates a rare and highly modified molecular scaffold. Both compounds showed strong cytotoxicity against A2780, SKOV3, HeLa, and HepG2 cancer cell lines, with IC_50_ values ranging from 3.9 ± 0.4 to 23 ± 1 µM, comparable to the positive control cisplatin (IC_50_ = 15 ± 1 µM). In addition, sucurchalasins A and B inhibited bacterial growth of *E. faecalis* and *B. subtilis*, with MIC values of 3.1 and 6.3 µg/mL, respectively [99]. Taken together, these cytochalasins show that small changes in the macrocyclic structure strongly influence their biological activity. Oxidation reactions such as epoxidation and hydroxylation can shift molecular shape, alter polarity, and change hydrogen‐bonding ability. As a result, they affect how these compounds interact with actin, which in turn influences actin polymerization and the cellular events that follow. The different cytotoxic profiles seen for the xylariachalasins (**103‐**‐**105**), cytochalasin SY derivatives (**106**–**108**), and rosellichalasins (**109**–**111**) suggest that even minor differences in macrocyclic structure and side‐chain substitution can significantly affect both potency and which cancer cells they target. In addition, the sulfur‐bridged cytochalasan–macrolide hybrids, sucurchalasins A and B (**112** and **113**), form an unusual structural class. Their higher conformational rigidity and the presence of extra interaction sites may strengthen binding to the target, which could help explain why they show a wider cytotoxic range than typical cytochalasins. Overall, these findings emphasize how macrocyclic structural features shape cytochalasin bioactivity and offer useful guidance for designing more effective actin‐targeting anticancer agents. Alkaloids made by endophytic fungi make up a very diverse group of nitrogen‐containing metabolites. They include indole alkaloids, diketopiperazines, quinazolines, cytochalasins, ergot alkaloids, and other heterocyclic scaffolds. Their pharmacological effects are strongly tied to specific structural characteristics, such as the arrangement of heterocyclic rings, prenylation, halogenation, oxidation patterns, and macrocyclization. Prenylated indole alkaloids usually show greater cytotoxic activity, likely because the added prenyl groups increase lipophilicity and improve binding to intracellular targets. By contrast, cytochalasins are well known for their powerful anticancer effects, mainly because they interfere with actin filament formation through their distinctive macrocyclic isoindolone framework. Oxygenated indole alkaloids, meanwhile, are more often linked to anti‐inflammatory roles and enzyme‐regulating functions. Among the metabolites covered here, asperdole D (**64**), aspertaichamide A (**80**), talarindigotin A (**90**), and chaepseubakerin A (**91**) stand out as particularly promising lead compounds, mainly due to their strong cytotoxicity and/or receptor‐selective biological activities. In general, the broad structural diversity of endophytic fungal alkaloids and the clear structure‐activity relationships highlight their strong potential as starting points for developing and improving anticancer treatments and other therapeutic agents.

### 2.4 Polyketides

**FIGURE 13:**
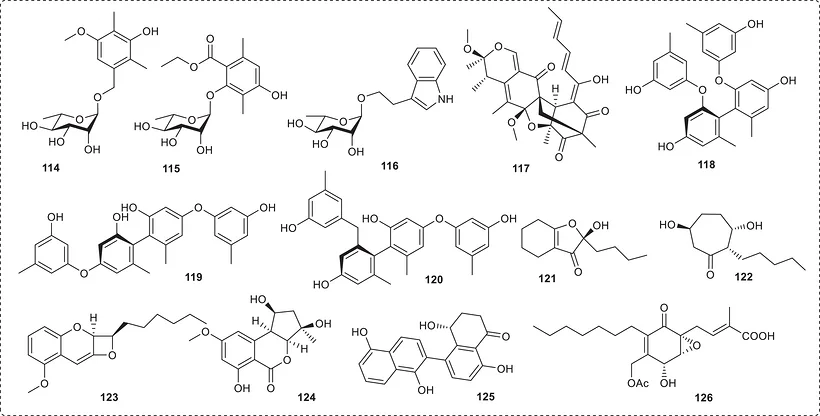
Structures of compounds **114‐**‐**126**.

Polyketides are among the largest and most structurally varied groups of specialized metabolites made by endophytic fungi, and they are an important source of natural products with biological activity [100]. Their remarkable structural diversity driven by the highly flexible biosynthetic pathways of polyketide synthases (PKSs) has enabled the identification of many compounds that show antimicrobial, anticancer, anti‐inflammatory, antioxidant, and enzyme‐inhibitory effects, highlighting their value in natural product chemistry and drug discovery [101]. Endophytic fungi from genera, such as including *Aspergillus, Penicillium, Fusarium, Colletotrichum*, and *Alternaria*, are well known for producing structurally diverse polyketides, many of which display strong activity against clinically and agriculturally important pathogens. This emphasizes their potential as leads for developing new therapeutic and agrochemical agents [102, 103, 104, 105, 106, 107]. Overall, polyketides from endophytes point to significant promise for pharmaceutical, agricultural, and biotechnological applications [108]. The endophytic fungus *B. dothidea* LE‐07, isolated from *L. chinense*, produced three previously unknown rhamnosylated metabolites: two polyketide‐derived phenolic rhamnosides, botryrhamnosides A (**114**) and B (**115**) (Figure 13), and a tryptophol alkaloid rhamnoside, botryrhamnoside C (**116**, Figure 13) [109]. Compounds **114‐**‐**116** showed antibacterial activity against *E. coli*, with MIC values between 8 and 10 µg/mL. In addition, compound **115** suppressed nitric oxide (NO) production in lipopolysaccharide (LPS)‐stimulated BV‐2 microglial cells, (IC_50_ = 28.30 ± 3.08 µM) while remaining minimally cytotoxic, with 81.32 ± 5.22% cell viability at 50 µM. These results suggest that its anti‐neuroinflammatory effect was unlikely to stem from general cytotoxicity [109]. The endophytic fungus *P. citrinum* ZY‐2, isolated from *P. asiatica* L., produced a previously undescribed sorbicillinoid derivative, citrosorbicillinol (**117**, Figure 13). Compound **117** exhibited strong osteogenic activity in a prednisolone‐induced zebrafish model of osteoporosis and also enhanced osteoblastic differentiation of MC3T3‐E1 cells at a concentration of 2 µM, suggesting its potential as a treatment for osteoporosis [110]. Three previously unknown phenoxybiphenyl derivatives, neohenbiphenylols A–C **(118–120,** Figure 13), were isolated from the endophytic fungus *N. kickxii* RT‐749, which is associated with *F. crenata* collected in Yamagata Prefecture, Japan [111]. All three compounds showed potent cytotoxicity against HL‐60 leukemia cells, with IC_50_ values of 3.7, 4.3, and 7.3 µM, respectively. Furthermore, compounds **118**–**120** displayed antibacterial activity against *B. subtilis*, with each compound having an MIC value of 25 µg/mL [111]. Another endophytic fungus, *Phoma* sp. YUD17001, was isolated from *G. elata* Blume, and found to produce two novel polyketides, phometides A (**121**) and B (**122**) (Figure 13) [112]. In biological testing, these compounds showed selective antimicrobial effects. Specifically, compound **121** inhibited the growth of *B. subtilis* and *S. aureus*, with MIC values of 64 and 8 µg/mL, respectively. Meanwhile, compound**122** suppressed the growth of *C. albicans*, with an MIC value of 16 µg/mL [112]. A previously undescribed polyketide, trichopsistide K (**123**, Figure 13), was isolated from the endophytic fungus *T. koningiopsis* WZ‐196, which is associated with *R. podantha* collected in China [113]. Compound **123** showed strong phytotoxic activity against *Arabidopsis thaliana*, with an IC_50_ value of 8.09 µM, indicating its potential use as a natural herbicidal lead [113]. The endophytic fungus Trichoderma aculeatus A884, isolated from *Aquilaria sinensis* collected in Guangdong, China, produced a previously undescribed polyketide, rubralactone aculeatone B (**124**,  Figure 13) [114]. Compound **124** inhibited nitric oxide (NO) production in LPS‐stimulated RAW 264.7 macrophages, with an IC_50_ value of 24.17 ± 1.78 µM. It also demonstrated acetylcholinesterase (AChE) inhibitory activity (IC_50_ = 18.43 µM) [114]. However, because cytotoxicity toward RAW 264.7 cells was not reported, the reduction in NO production should be viewed with caution.Liu et al. [115] reported a previously undescribed cladosporol derivative, named cytosporol C (**125**, Figure 13), isolated from the endophytic fungus *C. heveae* NSHSJ‐2, which is associated with the mangrove *S. caseolaris* collected in the Nansha Mangrove National Nature Reserve, Guangdong Province, China. Compound **125** showed cytotoxicity against the human breast cancer cell line MDA‐MB‐435, with an IC_50_ value of 7.7 µM [115].A previously undescribed ambuic acid derivative (**126**, Figure 13) was isolated from the fermentation broth of the endophytic fungus *P. chamaeropis*, which is associated with the medicinal plant *A. selengensis* [116]. The compound inhibited nitric oxide production in LPS‐stimulated RAW 264.7 macrophages, with an IC_50_ value of 19.7 ± 1.2 µM. It was also assessed for cytotoxicity against the A549, HepG2, and HeLa cell lines; however, since cytotoxicity data for RAW 264.7 macrophages were not provided, its anti‐inflammatory activity should be interpreted with caution [116].

**FIGURE 14:**
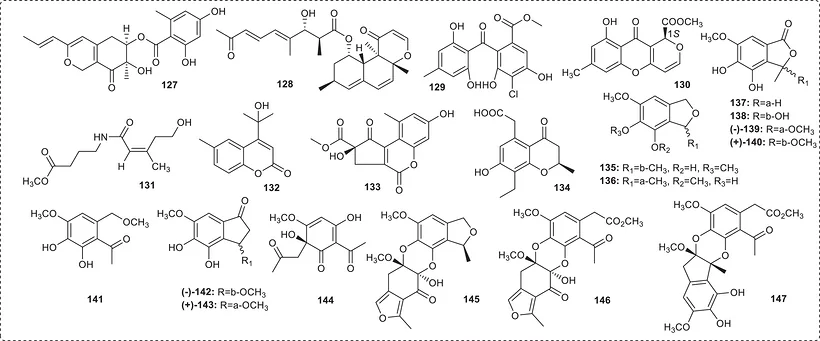
Chemical structures of compounds **127–147**.

The endophytic fungus *Aspergillus* sp. HMGh1‐1, which was collected from with the mangrove plant *C. manghas* L. in Guangxi, China, produced a phenanthrene‐derived polyketide that had never been reported before 12‐methyl entonaemin A (**127**, Figure 14) [117]. Compound **127** showed strong insecticidal activity against the Asian citrus psyllid *Diaphorina citri* Kuwayama reaching a corrected mortality rate of 90.97% [117]. Gu et al. [118] isolated japonidiene C (**128**, Figure 14) a calbistrin derivative that had not been described previously, from the endophytic fungus *A. japonicus* TE739D. This fungus was associated with the leaves of *N. tabacum* L. collected in Hubei Province, China. Compound **128** demonstrated herbicidal effects on *A. retroflexus* L. seedlings, with an IC_50_ value of 21.5 µg/mL [118]. A previously undescribed chloroisosulochrin derivative, demethyl‐chloroisosulochrin (**129**, Figure 14), was isolated from a *Penicillium* sp., living endophytically in *R. molle* collected in Guangxi Province, China [119]. Compound **129** showed moderate cytotoxicity against HepG2 liver cancer cells, with an IC_50_ value of 30.18 µM [119]. The endophytic fungus *A. versicolor* JGC‐G1 was isolated from the roots of *A. multiflora* collected in Liaoning Province, China. It produced a previously undescribed polyketide (**130**, Figure 14) that exhibited strong antifungal activity *C. albicans* (MIC of 3.125 µg/mL) [120]. Two bioactive polyketides chlovirescenols A (**131**) and B (**132**) (Figure 14), were isolated from the endophytic fungus *C. virescens* GLQ05, which is associated with *G. lucidum* collected in Guangxi, China [121]. At a concentration of 20 µM, both compounds lowered TNF‐α expression in LPS‐stimulated RAW 264.7 macrophages, based on RT‐qPCR results. Since macrophage cytotoxicity was not evaluated, these observations should be viewed as preliminary support for anti‐inflammatory activity [121]. Xiang et al. [122] reported two previously undescribed pyrone derivatives, ellagic acid C (**133**) and aspergone R (**134**) (Figure 14), isolated from the endophytic fungus *Diaporthe* sp. CB10100. This fungus was recovered from the roots of *S. acutum* collected in Hunan Province, China. Both compounds showed weak antimicrobial activity against methicillin‐resistant Staphylococcus aureus (MRSA), Klebs iella pneumoniae, and Mycolicibacterium smegmatis, with MIC values above 64 µg/mL. In addition, they reduced the expression of inducible nitric oxide synthase (iNOS) and cyclooxygenase‐2 (COX‐2) in LPS‐stimulated RAW 264.7 macrophages, indicating possible anti‐inflammatory effects. However, because no data on macrophage cytotoxicity were provided, no firm conclusions can be made about their anti‐inflammatory mechanism [122]. The endophytic fungus *O. cirsii* LZU‐1509, isolated from the medicinal plant *A. lacteal*, yielded thirteen previously undescribed polyketides. These included three benzofuran derivatives (**135**‐**137**, Figure 14), one benzofuran racemate (**138**), two pairs of polyketide enantiomers [(±)‐**139** and (±)‐**140**], two acetophenone derivatives (**141** and **144**) (Figure 14), and three aromatic polyketides containing 1,4‐dioxane (**145**‐**147**, Figure 14) [123]. All of the metabolites inhibited the growth of HepG2 and HT‐1080 tumor cell lines at 20 µM. Furthermore, compounds **137** and **139** protected hydrogen peroxide‐ treated neuron‐like PC12 cells from oxidative injury, suggesting they may have strong antioxidant activity [123].

**FIGURE 15:**
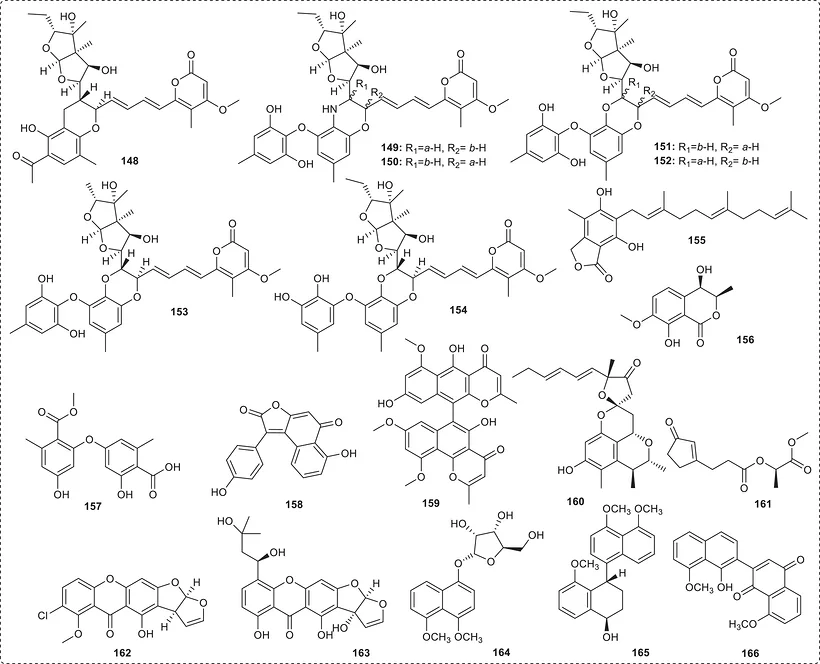
Chemical structures of compounds **148–166**.

Seven previously undescribed polyketide‐derived mycotoxins, asteltoxins A‐O (**148**‐**154**, Figure 15), were isolated from the endophytic fungus *P. bulbillosa* KNI755, which was associated with *P. lingua* collected in China [124]. These compounds showed significant immunosuppressive effects, as they inhibited concanavalin A (ConA)‐driven T‐cell proliferation and lipopolysaccharide (LPS)‐induced B‐cell proliferation. Their EC_50_ values ranged from 9.2 to 26.0 µM for T‐cell inhibition and from 6.3 to 27.0 µM for B‐cell inhibition. In addition, compound **148** also exhibited cytotoxic activity against HCT116, HT29, and A549 cells, with IC_50_ values of 10.0 ± 0.5 µM, 17.0 ± 2.0 µM, and 25.0 ± 1.0 µM, respectively. Meanwhile, compound **149** showed selective cytotoxicity toward HCT116 cells only, with an IC_50_ value of 22.0 ± 2.0 µM [124]. The endophytic fungus *Penicillium* sp. MNP‐HS‐2, isolated from *M. communis*, produced a previously unreported polyketide named, mycophenolene A (**155**, Figure 15) [125]. Compound **155** displayed strong cytotoxicity against KB3.1 cells, with an IC_50_ of 8.3 µM, and it also showed antibacterial activity against *S. aureus*, with a MIC of 2.1 µg/mL [125]. A previously undescribed isocoumarin derivative (**156**, Figure 15) was isolated from the endophytic fungus *P. neosporulosa* VDB39, which was associated with *V. dunalianum* collected in Yunnan, China [126]. Compound **156** exhibited broad‐spectrum antifungal activity against several phytopathogens, including *B. cinerea, F. solani, F. graminearum, F. oxysporum*, and *A. brassicicola*, with MIC values ranging from 7.81 to 15.63 µg/mL [126]. The endophytic fungus, *Aspergillus* sp. GXNU‐DY2‐12, which was isolated from the leaves of the mangrove plant *A. ilicifolius* L. collected in China, was found to produced a previously unknown diphenyl ether derivative called pseudopithoether C (**157**, Figure 15) [127]. Compound **157** showed strong acetylcholinesterase (AChE) inhibitory activity, with an IC_50_ of 1.02 µM, and it also displayed marked insecticidal effects against *D. citri Kuwayama*, leading to an 88% mortality rate [127]. Similarly, the endophytic fungus *G. siamensis* isolated from *B. nutans* collected in Phetchabun Province, Thailand, produced a previously undescribed compound: 6‐hydroxy‐1‐(4‐hydroxyphenyl)naphtho[2, 1‐b]furan‐2, 5‐dione (**158**, Figure 15) [128]. Compound **158** was selectively cytotoxic to HT‐29 colon cancer cells (IC_50_ = 11.31 ± 2.13 µg/mL) and showed moderate activity against PC‐3 prostate cancer cells (IC_50_ = 24.62 ± 0.26 µg/mL) [128]. A novel naphtho‐γ‐pyrone dimer, asperosperma A (**159**, Figure 15), was isolated from the endophytic fungus *A. niger*, associated with the stems of *C. flavida* [129]. Compound **159** demonstrated outstanding cytotoxic activity against H460 and 4T1 cancer cell lines, with IC_50_ values of 0.37 ± 0.06 µM and 2.04 ± 0.79 µM, respectively. It also exhibited broad‐spectrum antibacterial activity against S. aureus, Escherichia coli, Pseudomonas aeruginosa, and methicillin‐resistant S. aureus (MRSA), generating inhibition zones ranging from 20.0 to 29.5 mm [129]. The endophytic fungus *A. cristatus, isolated from the stems of N. tabacum*, produced a previously undescribed polyketide, aspercitrininone A (**160**, Figure 15) [130]. Compound **160** showed antibacterial activity against Gram‐positive bacteria, including *S. aureus*, a clinical *S. aureus* isolate*, B. subtilis*, and *E. faecalis*, with MIC values ranging from 22.5 ± 2.3 to 48.3 ± 6.5 µg/mL. No activity was observed against *E. coli* [130]. A previously undescribed trichodenone derivative (**161**, Figure 15) was isolated from the endophytic fungus *T. hamatum* B‐3, which is associated with *B. purpurascens* collected from Mount Emei, China [131]. Compound **161** displayed moderate antifungal activity against *F. oxysporum*, with inhibition rate 51.0 ± 3.4%, while showing weaker activity against the other tested phytopathogenic fungi [131]. The endophytic fungus *A. puniceus*, isolated from *E. chinense* collected in Hubei Province, China, produced two xanthone derivatives: austocystin S (**162**) and austocystin R (**163**) (Figure 15) [132]. Both compounds strongly inhibited protein tyrosine phosphatase 1B (PTP1B), with IC_50_ values of 2.89 µM and 0.78 µM, respectively, suggesting strong antidiabetic potential. In addition, compounds **162** and **163** showed high cytotoxicity against MDA‐MB‐231 breast cancer cells, with IC_50_ values of 1.45 µM and 1.28 µM, respectively [132]. Another endophytic fungus, *D. eschscholzii* MCZ‐18, isolated from the mangrove plant *C. tagal*, produced three previously undescribed naphthalene derivatives, dalesconosides A‐C (**164**‐**166**, Figure 15) [133], activities of all isolated compounds were evaluated against *P. aeruginosa, E. faecalis*, methicillin‐resistant *S. aureus* (MRSA), *E. coli*, and *C. albicans*. Compound **164** exhibited broad‐spectrum antibacterial activity against *P. aeruginosa*, *E. coli*, *E*. faecalis and methicillin‐resistant S. aureus (MRSA), with MIC values as low as 6.25 µg/mL. Compound **165** had strong activity against *E. coli*, (MIC = 12.5 µg/mL), whereas, compound **166** showed moderate activity against *P. aeruginosa*, (MIC = 25 µg/mL) [133]. In contrast, monomeric xanthones like austocystins S (**162**) and R (**163**) show strong inhibition of protein tyrosine phosphatase 1B (PTP1B). This suggests that their activity depends greatly on the specific oxygenation patterns and the orientation of their substituents. The conserved xanthone core, along with carefully placed hydroxyl and methoxy groups, likely supports beneficial hydrogen bonding and hydrophobic interactions within the PTP1B catalytic site, which in turn boosts inhibitory strength. Supporting this idea, austocystin R (**163**) inhibited PTP1B more effectively (IC_50_ = 0.78 µM) than austocystin S (**162**) (IC_50_ = 2.89 µM), indicating that even small structural changes can significantly affect target binding. However, both compounds also showed pronounced cytotoxicity toward MDA‐MB‐231 cells (IC_50_ = 1.28 and 1.45 µM, respectively), pointing to relatively limited selectivity between enzyme inhibition and antiproliferative effects. Overall, the biological performance of fungal xanthones is largely controlled by their structural framework and the placement of functional groups. While dimerization may strengthen target interactions and increase biological potency, it may also raise molecular hydrophobicity and lead to more nonspecific cytotoxicity. On the other hand, thoughtful optimization of oxygenation patterns in monomeric xanthones could help improve enzyme selectivity. Taken together, these structure‐activity relationships highlight the importance of deliberate structural modification in the development of xanthone‐based therapeutics.

**FIGURE 16:**
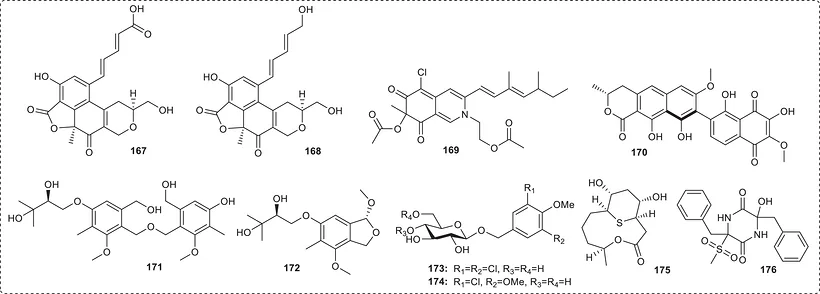
Structures of compounds **167**–**176**.

The endophytic fungus *T. pinophilus* WRH‐1, which was isolated from *P. frutescens* (L.) Britt. frutescens collected in Hangzhou, China, produced two azaphilone metabolites, that had not been reported before: acetosellins A (**167)** and B (**168)** (Figure 16) [134]. Both compounds showed moderate cytotoxic effects on H23 human lung cancer cells, with IC_50_ values of 14.10 µM and 14.65 µM, respectively, compared with the positive control sorafenib (IC_50_ = 2.40 µM). In addition, compound **167** inhibited the growth of *S. aureus* and *B. subtilis*, with MIC values of 64 and 32 µg/mL, respectively, while compound **168** displayed antibacterial activity against both strains, with MIC values of 64 µg/mL. These metabolites also promoted bone formation in a zebrafish model of osteoporosis, achieving effective concentrations of 4 µM and 2 µM, respectively, suggesting strong potential as anti‐osteoporotic agents [134]. Another endophytic fungus, *D. pseudooculi* HHUF 30617, obtained from the leaves of the mangrove *Sonneratia* sp. collected in Guangzhou, China, produced a previously undescribed polyketide: N‐acetoxyethyl‐3‐(3,5‐dimethyl‐1,3‐heptadienyl)‐5‐chloro‐7‐methyl‐7‐acetoxy‐6,8‐(2H,7H)‐isoquinolinedione (**169**, Figure 16) [135]. At 6.25 µM, compound **169** suppressed CuSO_4_‐induced inflammatory responses in zebrafish larvae, indicating its anti‐inflammatory potential in this in vivo setting [135]. A previously undescribed polyketide derivative, xanthoradone D (**170**, Figure 16), was isolated from the endophytic fungus *A. aureus* HJ‐7, which is associated with *A. mamillata* Hance collected in Guangxi, China [136]. Compound **170** demonstrated strong antibacterial activity against *S. aureus* and *X. axonopodis* pv. citri, with MIC values of 6.25 µg/mL, and also showed antifungal powerful activity against *C. miyabeanus* and *C. paradoxa*, with MIC values of 3.125 µg/mL [136]. A pair of previously unknown phthalate‐related metabolites, alternyxtiols B (**171**) and G (**172**) (Figure 16), were isolated from the endophytic fungus *Alternaria* sp. YUD24001, which is associated with *A. hemsleyanum* collected in Sichuan Province, China [137]. Both compounds showed antifungal effects against the phytopathogens *F. solani, A. panax*, and *E. nigrum*, with MIC values of 16, 32, and 16 µg/mL respectively [137]. Two previously undescribed metabolites, fuscoposides **173** and **174** (Figure 16), were isolated from the mangrove‐derived endophytic fungus *Fuscoporia* sp. A2A6, obtained from leaf tissues collected in Penang, Malaysia [138]. Compound **173** displayed moderate neuroprotective activity in HT‐22 mouse hippocampal neuronal cells at 6.0 µM. On the other hand, compound **174** decreased cyclooxygenase‐2 (COX‐2) expression in BV2 microglial cells at 5.0 µM, suggesting possible anti‐inflammatory effects. However, because cytotoxicity toward BV2 cells was not reported, this finding should be treated cautiously [138]. Li et al. [139] reported two previously undescribed sulfur‐containing polyketides, ascomycotol A (**175**, Figure 16) and ascomycotol B (**176**, Figure 16), isolated from *Ascomycota* sp. GLQ01 and *T. harzianum* GLQ03. Both strains were associated with Ganoderma spp. collected in Guangxi Province, China. Ascomycotol A contains a rare 4‐oxa‐12‐thiabicyclo[7.3.1]tridecane skeleton. Both compounds inhibited inflammatory responses at 20 µM and provided protective effects in a renal fibrosis model, highlighting their promise as lead candidates for developing anti‐inflammatory and anti‐fibrotic agents [139].

**FIGURE 17:**
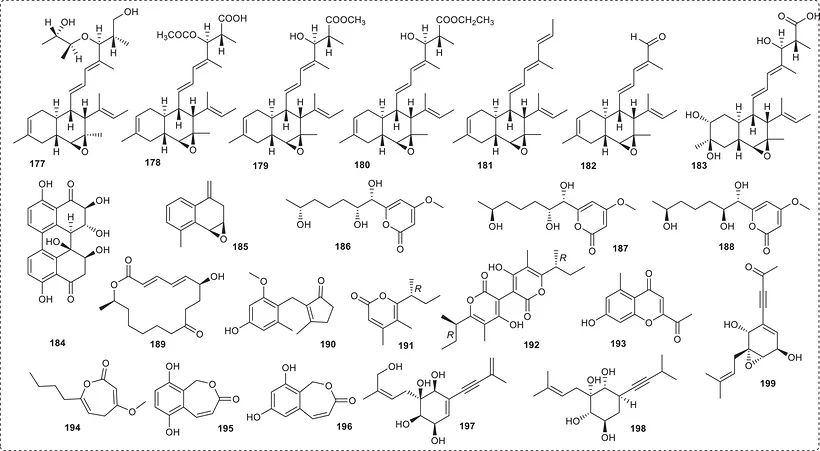
Chemical structures of compounds **177–199**.

Shi et al. [108] studied the secondary metabolites produced by the endophytic fungus *F. asiaticum* QA‐6, which they from the medicinal plant *Artemisia argyi* collected in Hubei Province, China. From this fungus, they identified seven polyketide derivatives that had not been reported before, named fusariumtides A‐G (**177**‐**183**, Figure 17). These compounds showed moderate to strong antimicrobial effects against a range of aquatic pathogenic bacteria, including *Aeromonas hydrophila*, *E. ictaluri, E. tarda, Micrococcus luteus, P. aeruginosa, V. alginolyticus, V. harveyi, V. parahaemolyticus*, and *V. vulnificus*. Their MIC values were in the range of 1–64 µg/mL [108]. A previously undescribed perylenequinone derivative (**184**, Figure 17) was isolated from the endophytic fungus *A. alstroemeriae*, which is linked to *C. roseus* collected in Yunnan Province, China [140]. Compound **184** displayed strong antimicrobial activity against *S. aureus* and *C. albicans*, with MIC values of 3.125 and 1.5625 µg/mL, respectively [140]. The endophytic fungus *T. purpureogenus*, collected from the leaves of *R. molle* in Xing'an, China, produced a previously unreported epoxynaphthalenone derivative (**185**, Figure 17) [141]. Compound **185** showed moderate xanthine oxidase (XOD) inhibition, inhibitory activity, achieving 40.1% inhibition at 10 µM, compared with 99.3% inhibition produced by the positive control, febuxostat [141]. Three previously undescribed polyketide‐derived pyrones (**186**‐**188**, Figure 17) were obtained from the mangrove‐associated endophytic fungus *Phomopsis* sp. DHS‐11, obtained which was isolated from *R. mangle* collected in Hainan, China [142]. Compounds **186** and **187** showed potent cytotoxicity against HepG2 hepatocellular carcinoma cells, with IC_50_ values of 4.66 ± 0.35 and 6.07 ± 0.48 µM, respectively both exceeding the activity of the positive control, 5‐fluorouracil (IC_50_ = 21.69 ± 9.11 µM). Compound **188** exhibited moderate cytotoxic activity against HeLa cervical cancer cells, with an IC_50_ of 20.20 ± 4.93 µM, whereas doxorubicin had an IC_50_ value of 0.95 ± 0.61 µM [142]. A previously undescribed polyketide, trichodone F (**189**, Figure 17), was isolated from the endophytic fungus *T. protrudens*, which is associated with the stems of *H. tiliaceus* [143]. Compound **189** showed moderate cytotoxic activity against HepG2 hepatocellular carcinoma cells, with an IC_50_ of 22.7 µM, but displayed little or no activity against MCF‐7 breast cancer cells [143]. Bai et al. [144] examined the secondary metabolites from the endophytic fungus *P. brefeldianum* F4a, which they isolated from the roots of *H. cordata* Thunb. collected in Hunan, China. From this fungus, they identified a previously unknown polyketide, peniorcinol D (**190**, Figure 17). Compound **190** showed α‐glucosidase inhibitory activity, with an EC_50_ of 29.8 ± 9.4 µM, suggesting moderate potential for inhibiting the enzyme. In another study, the endophytic fungus *Fusarium* sp. HC20, isolated from the roots of *R. rosea* L. collected in Tibet, China, produced three previously undescribed polyketides: fusaritricins E‐G (**191**‐**193**, Figure 17) [145]. All three compounds inhibited the growth of *C. albicans*, with MIC values between 12.5 to 25 µg/mL, though they were less active against *A. niger* R330 [145]. A previously undescribed polyketide, xylacurtin A (**194**, Figure 17) was also reported, isolated from the endophytic fungus *X. curta* YSJ‐5 associated with the medicinal plant *A. zerumbet* collected in China [146]. Compound **194** demonstrated strong antibacterial effects against both *S. aureus* and methicillin‐resistant S. aureus MRSA, with an MIC value of 6.3 µg/mL [146]. Two previously undescribed polyketides, aculeatides J (**195)** and K (**196)** (Figure 17), were isolated from the endophytic fungus *A. aculeatus* [147]. Both compounds showed strong herbicidal activity, with IC_50_ values of 3.01 ± 0.40 µM and 3.33 ± 0.52 µM, respectively, underscoring their potential as natural phytotoxic agents [147].A related compound, aspergienyne C (**197**, Figure 17), a previously undescribed diisoprenyl cyclohexene‐type meroterpenoid, was isolated from the mangrove‐derived endophytic fungus *Aspergillus* sp. GXNU‐Y65, associated with *K. candel*, collected in Beihai, China [148]. In AML12 hepatocytes, compound **197** reduced palmitic acid‐ and oleic acid‐induced triglyceride buildup, indicating possible protective effects against non‐alcoholic steatohepatitis (NASH) [148]. Another mangrove‐derived endophytic fungus, *Aspergillus* sp. GXNU‐Y85, was isolated from the fruits of *K. candel* collected in Beihai, China, produced a previously undescribed diisoprenyl cyclohexene‐type meroterpenoid called aspergienyne N (**198**, Figure 17) [149]. Compound **198** showed moderate cytotoxic activity HeLa cervical cancer cells, with an IC_50_ value of 28.4 µM [149]. Finally, an endophytic fungus closely related to these, *Aspergillus* sp. GXNU‐QG1a, isolated from *K. candel* collected in Guangxi, China, produced another previously undescribed diisoprenyl cyclohexene‐type meroterpenoid, biscognienyne M (**199**, Figure 17) [150]. Compound **199** displayed significant cytotoxicity against A2780 ovarian carcinoma cells and MIA PaCa‐2 pancreatic cancer cells, with IC_50_ values of 6.81 µM and 15.40 µM, respectively, pointing to promising anticancer potential [150]. Polyketides are the most abundant and structurally varied metabolites made by endophytic fungi. They include compounds such as anthraquinones, xanthones, chromones, azaphilones, benzopyranones, benzophenones, isocoumarins, and pyrones. Their biological effects are largely determined by how the molecules are modified for example through hydroxylation, oxidation, halogenation, methylation, and dimerization. In general, polyketides with many hydroxyl groups tend to show strong antioxidant and anti‐inflammatory activity. By contrast, highly oxygenated anthraquinones and xanthones are more often linked to powerful cytotoxic effects. Halogenated and dimeric polyketides frequently have stronger antimicrobial properties, which is thought to result from their increased lipophilicity and better interactions with biological targets. Overall, the exceptional structural diversity of fungal polyketides, along with clear structure‐activity relationships, makes them a highly promising reservoir of lead compounds for developing anticancer, antimicrobial, and anti‐inflammatory drugs.

#### 2.4.1 Lactones

**FIGURE 18:**
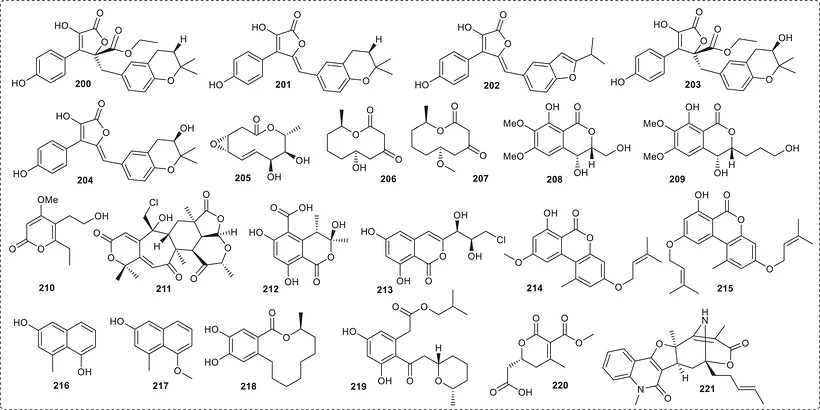
Chemical structures of compounds **200–221**.

The endophytic fungus *A. terreus* AArEF2, which was isolated from the leaves of *A. arborescens* L., produced five previously undescribed butenolide metabolites: asperterreunolides A‐E (**200**‐**204**, Figure 18) [151]. The cytotoxic effects of these compounds were tested on the A‐431, C4‐2B, and MDA PCa 2b human cancer cell lines using the MTT assay. All five metabolites showed strong cytotoxicity, with IC_50_ values between 3.72 and 6.27 µM across the cell lines examined. For comparison, the positive control doxorubicin had IC_50_ values of 0.097, 0.094, and 0.033 µM, respectively [151]. A previously undescribed lactone, multiplolide I (**205**, Figure 18), was isolated from the endophytic fungus *Aspergillus* sp. GXNU‐HLC1Y2‐2, which is associated with *H. littoralis* Dryand [152]. Compound **205** demonstrated potent insecticidal activity against *D. citri* Kuwayama, producing a corrected mortality rate of 93.96% [152]. The endophytic fungus *P. restrictum*, collected from the roots of *P. tunicoides* in Yunnan, China, produced two previously undescribed 10‐membered lactones, penicirestricols A (**206**) and B (**207**) (Figure 18) [83]. Both compounds suppressed the growth of the phytopathogenic fungi *A. tenuissima* and *F. asiaticum*, with MIC values ranging from 1 to 32 µg/mL [83]. Three lactone‐related metabolites, including two previously undescribed isocoumarins (**208** and **209**), and an α‐pyranone derivative (**210**) (Figure 18), were isolated from the mangrove‐derived endophytic fungus *E. rostratum* NS‐KS‐3, which is associated with *K. candel* (L.) Druce collected in Guangzhou, China [153]. All three compounds inhibited α‐glucosidase, with IC_50_ values of 0.093, 0.168, and 0.250 mM, respectively, suggesting strong antidiabetic potential [153]. A previously undescribed meroterpenoid, amestolknoid C (**211,** Figure 18), was isolated from the endophytic fungus *T. amestolkiae* SCNU‐F0041, obtained from the mangrove plant *K. obovata* collected in Guangdong Province, China [154]. Compound **211** showed significant antiviral activity against SARS‐CoV‐2, with an EC_50_ value of 2.50 ± 0.42 µM. It also decreased nitric oxide production in lipopolysaccharide‐stimulated RAW 264.7 macrophages (IC_50_ = 4.10 µM). However, since reducing nitric oxide by itself does not confirm a clear anti‐inflammatory mechanism and the observed effect could be affected by cytotoxicity if cell viability is not verified these results should be interpreted carefully [154]. A previously undescribed isocoumarin derivative, (±)‐isothielavic acid (**212**, Figure 18), was isolated from the endophytic fungus *T. terrestris* YB4, associated with the rhizomes of *S. mairei* collected in the Kunming Botanical Garden, China [155]. Compound **212** exhibited antibacterial activity against *B. subtilis*, *P. aeruginosa*, and *E. coli*. producing inhibition zones of 7, 9, and 10 mm, respectively [155]. A previously unreported isocoumarin derivative, desmethyldichlorodiaportinol A (**213**, Figure 18), was isolated from the cell culture of the endophytic fungus *D. arengae* M2. This fungus was obtained from *C. oleifera* collected in Hainan Province, China. Its antimicrobial activity was assessed against a range of pathogenic microorganisms, including *S. aureus, E. coli, P. aeruginosa, B. subtilis*, and methicillin‐resistant *S. aureus* (MRSA). Compound **213** showed strong antimicrobial effects against *S. aureus*, MRSA, and *B. subtilis*, with a minimum inhibitory concentration (MIC) of 16 µg/mL [156]. The endophytic fungal strain *A. malorum* FL39, isolated from *M. bontioides* collected on the Leizhou Peninsula, China, produced two previously undescribed isocoumarin derivatives, alternariethers A and B (**214** and **215**, Figure 18). Their antimicrobial properties were tested in vitro against the phytopathogenic fungi *C. musae, F. oxysporum*, and *F. graminearum*, as well as against the human pathogenic bacteria, *E. coli*, and *S. aureus*. Alternariether A (**214)** displayed moderate antimicrobial activity against *C. musae* and *E. coli*, with an MIC of 50 µg/mL. In contrast, alternariether B (**215)** showed antibacterial activity against *E. coli*, with an MIC of 50 µg/mL [157]. Two polyketide‐derived naphthalene metabolites, protoventurenes A and B (**216** and **217**, Figure 18), were isolated from the ethyl acetate fraction of the endophytic fungus *Protoventuria* sp. E‐055. This fungal strain was collected from leaf tissues of *V. macrocarpon* in Ontario, Canada. When tested at a dose of 100 µg (equivalent to 20 µL of a 5 mg·mL^−^^1^ solution), both compounds showed marked antifungal activity against the phytopathogen *B. cinerea*. Furthermore, compound **217** exhibited significant inhibitory activity against *C. gloeosporioides* at the same concentration [158]. Penicimenolide G (**218**, Figure 18), a metabolite that had not been reported previously, was obtained from the fermentation broth of the endophytic fungus *A. giganteus*. This fungus was found living in the leaf tissue of *C. carandas*, which was collected from a medicinal plant garden in Bangladesh. In vitro testing showed that compound **218** had antibacterial activity against *B. cereus*. It produced an inhibition zone of 9 mm, respectively, demonstrating measurable antibacterial effects under the experimental conditions [159]. When the mangrove‐derived endophytic fungus *P. brocae* MA‐231 was co‐cultured with the phytopathogenic fungus *C. spicifera* QA‐26, it resulted in the formation of a previously undescribed curvularin derivative, curvulone C (**219**, Figure 18). Both fungal strains were originally isolated from *A. argyi* collected in Hubei Province, China. Compound **219** was evaluated for antibacterial activity against a panel of pathogenic bacteria, including *A. hydrophila, E. coli, M. luteus, P. aeruginosa, V. anguillarum, V. harveyi, V. parahaemolyticus*, and *V. vulnificus*. The compound inhibited the growth of *V. parahaemolyticus* and *V. vulnificus*, with an MIC of 64 µg/mL [160]. The endophytic fungus *T. purpureogenus*, isolated from the leaves of *R. molle* collected in Xing'an, China, produced a previously undescribed pyrone derivative called pyroneaceacid (**220**, Figure 18). Compound **220** was assessed for its inhibitory activity against xanthine oxidase (XOD), an enzyme essential for uric acid biosynthesis. At 10 µM, compound **220** inhibited XOD activity by 69.9%, while the reference inhibitor febuxostat produced 99.3% inhibition [141]. In another investigation, the endophytic fungus, *Penicillium* sp. NX‐S‐6 was found to produce three previously undescribed bioactive secondary metabolites, including a sorbicillinoids (**221**. Compound **221** strongly reduced interleukin‐6 (IL‐6) production in lipopolysaccharide (LPS)‐stimulated RAW264.7 macrophages, with an IC_50_ of 13.51 ± 6.70 µM [161]. Lactones made by endophytic fungi form a highly diverse set of secondary metabolites, featuring α,β‐unsaturated γ‐lactones, δ‐lactones, butenolides, macrolactones, and fused polycyclic lactones. Their biological effects are mainly shaped by structural factors such as the α,β‐unsaturated carbonyl group, how extensively the rings are fused, the oxidation pattern, and the kind and location of functional substituents. In particular, the electrophilic α,β‐unsaturated lactone portion often boosts cytotoxic, antimicrobial, and anti‐inflammatory activities because it can function as a Michael acceptor. At the same time, substituents such as hydroxyl, methoxy, and epoxy groups can further fine‐tune potency and improve target selectivity. Moreover, macrocyclic and more highly oxygenated lactones tend to show wider pharmacological effects, likely due to better molecular recognition and stronger interactions with biological targets.Among the metabolites discussed, compounds **208‐**‐**210** showed the strongest α‐glucosidase inhibitory activity, with IC_50_ values of 0.093, 0.168, and 0.250 mM, respectively. This suggests they could serve as promising starting points for developing antidiabetic therapies. Overall, the impressive structural diversity of fungal lactones and their clear structure‐activity relationships highlight their usefulness as privileged scaffolds for discovering and optimizing antimicrobial, anticancer, anti‐inflammatory, and enzyme‐targeted treatments.

#### 2.4.2 Ketones

**FIGURE 19:**
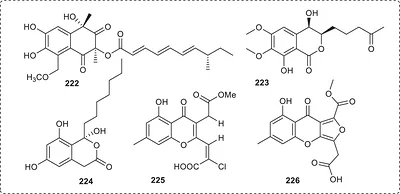
Structures of compounds **222‐**‐**226**.

A previously unreported bicyclic tetralone, tanicutone D (**222**, Figure 19), was isolated from the endophytic fungus *T. funiculosus* TF37. This fungus was obtained from fresh leaves of the medicinal plant *C. roseus* (L.). The biological activity of compound **222** was assessed in vitro using a range of pathogenic microorganisms and human cancer cell lines. For the antimicrobial tests, the panel included *S. aureus, S. cerevisiae, C. albicans*, and methicillin‐resistant *S. aureus* (MRSA). Cytotoxicity was tested against A549, NCI‐H1944, HepG2, and HeLa. Compound **222** showed broad‐spectrum antimicrobial effects with MIC values of 5.2, 10.0, 21.6, and 10.4 µg/mL against *S. aureus, S. cerevisiae, C. albicans*, and MRSA, respectively. In addition, it produced cytotoxic effects on all four cancer cell lines, with IC_50_ values of 20.18 ± 0.16 for A549, 19.19 ± 0.19 µM for NCI‐H1944, 19.37 ± 0.19 µM for HepG2, and 22.87 ± 0.23 µM for HeLa cells [162]. Two polyketide‐derived metabolites, exserone B (**223**) and cytosporone F (**224**) (Figure 19), were isolated from the ethyl acetate extract of a mangrove‐derived endophytic *Aspergillus* species collected in Guangxi, China. Both compounds showed notable insecticidal activity against the citrus psyllid, *Diaphorina citri*. At a dose of 1000 mg/kg, exserone B (**223**) and cytosporone F (**224**) caused mortality rates of 95.4% and 93.7%, respectively, highlighting their strong insecticidal potential [163]. The fungal strain *B. eleusines*, isolated from fresh potato tissues collected near Yunnan Province, China, generated several previously unknown secondary metabolites, including chromone derivatives eleusineketones A and B (**225** and **226**) (Figure 19). Their cytotoxic effects were evaluated using the human breast cancer cell line MDA‐MB‐231. Compounds **225** and **226** displayed moderate cytotoxicity, IC_50_ values of 14.48 and 17.99 µM, respectively [164]. Ketones produced by endophytic fungi form a highly varied group of secondary metabolites, and their biological effects are largely shaped by features such as the carbonyl group, conjugation, ring fusion, and hydroxylation patterns. α,β‐Unsaturated ketones often show stronger cytotoxic and antimicrobial activity because their electrophilic enone portion is particularly reactive, while hydroxylated ketones are more frequently linked to anti‐inflammatory and antioxidant effects. Of the metabolites reported, compound **222** showed the strongest antimicrobial activity overall. Taken together, the structural diversity of fungal ketones and their structure–activity relationships highlight their promise as useful starting points for developing antimicrobial, anticancer, and anti‐inflammatory drugs.

#### 2.4.3 Chromone Compounds

**FIGURE 20:**
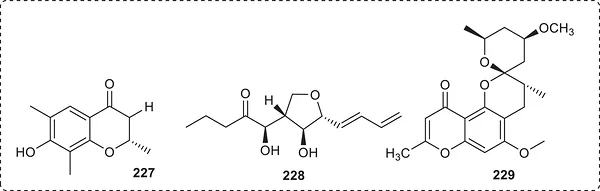
Structures of compounds **227–229**.

Two polyketide‐derived metabolites that had not been described previously namely, a chromone derivative (**227**) and a tetrahydrofuran‐containing analogue (**228**) (Figure 20), were isolated from a cell culture of the endophytic fungus *Chaetomium* sp. UJN‐EF006. This fungal strain was obtained from the leaves of *V. bracteatum* collected in Shandong Province, China. The anti‐inflammatory effects of compounds **227** and **228** were assessed in vitro using lipopolysaccharide (LPS)‐stimulated RAW 264.7 macrophages, which is a commonly used cellular model for studying inflammatory responses. At a concentration of 4 µM, both metabolites significantly suppressed LPS‐induced nitric oxide (NO) production, indicating that they may be able to influence inflammatory signaling under the conditions tested [165]. In addition, a structurally distinctive chromone derivative containing the rare [6]‐spiroketal moiety, chaetovirexylarione (**229**, Figure 20), was isolated from the ethyl acetate fraction obtained after co‐cultivating the endophytic fungi *C. virescens* and *X. grammica*. Both fungal strains were associated with *S. glabra* Roxb., collected in Guizhou Province, China. Compound **229** showed strong chemosensitizing activity in paclitaxel (PTX)‐resistant SW620/AD300 cells, enhancing PTX sensitivity by roughly 45‐fold. The metabolite was also identified as a potent inhibitor of P‐glycoprotein (P‐gp), further supporting its potential as a chemosensitizer capable of overcoming multidrug resistance in cancer cells [166].Chromones obtained from endophytic fungi have a shared, conserved 4H‐chromen‐4‐one core, and their biological effects are mainly shaped by hydroxylation, methoxylation, prenylation, and other oxygen‐bearing substitutions. In general, hydroxylated chromones tend to show stronger antioxidant and anti‐inflammatory activity, while methoxy‐ and prenyl‐substituted variants often display greater cytotoxic and antimicrobial effects. Of the metabolites reported so far, chaetovirexylarione (**229**) is the most promising candidate, owing to its distinctive [6]‐spiroketal chromone scaffold and its strong capacity to reverse paclitaxel resistance by inhibiting P‐glycoprotein. Overall, the wide structural variety of fungal chromones and the clear structure–activity relationships they exhibit suggest that these compounds could serve as useful lead scaffolds for developing antimicrobial, anticancer, anti‐inflammatory, and antioxidant drugs.

#### 2.4.4 Α‐Pyrone Compounds

**FIGURE 21:**
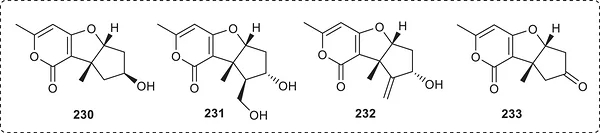
Structures of compounds **230–233**.

Four previously undescribed α‐pyrone derivatives, peniapyrones F‐I (**230**–**233**, Figure 21), were isolated from the endophytic fungus *P. brefeldianum* F4a. This fungus was associated with *H. cordata*, collected in Hunan Province, China. Structurally, these metabolites feature an unusual cyclopenta[4,5]furo[3,2‐c]pyran‐1‐one framework, formed by a fused 6/5/5‐tricyclic ring system. The cytotoxic activity of the isolated compounds was assessed in vitro using three human cancer cell lines: AsPC‐1, CRL‐2234, and MCF‐7. Among the four metabolites, peniapyrone F **(230),** and peniapyrone I **(233)** showed strong cytotoxicity toward the tested cell lines. Compound **230** produced IC_50_ values of 1.5 ± 0.1 µM against AsPC‐1 cells and 4.6 ± 0.7 µM against MCF‐7 cells, whereas compound **233** gave corresponding IC_50_ values of 2.9 ± 0.2 and 9.9 ± 0.9 µM, respectively. Overall, these findings highlight the α‐pyrone scaffold especially the cyclopenta[4,5]furo[3,2‐c]pyran‐1‐one framework as a promising structural motif for identifying cytotoxic fungal metabolites. Compound **231** showed moderate cytotoxic activity against CRL‐2234 and MCF‐7 cell lines, with IC_50_ values of 8.8 ± 0.8 and 5.5 ± 0.6 µM, respectively. Additionally, compound **232** displayed potent cytotoxic activity against all tested cancer cell lines, with IC_50_ values of 1.7 ± 0.2, 2.7 ± 0.3, and 0.2 ± 0.01 µM, respectively [167].

α‐Pyrones obtained from endophytic fungi are defined by a six‐membered unsaturated lactone ring. Their biological effects are mainly shaped by hydroxylation, alkyl or prenyl substitution, and the extent of conjugation. In general, hydroxylated α‐pyrones tend to show stronger antioxidant and antimicrobial activities, while lipophilic alkyl‐ and prenyl‐substituted derivatives often have greater cytotoxicity. Among the metabolites that have been reported, peniapyrone H (232) displayed the highest potency, with submicromolar to low‐micromolar cytotoxic effects against three cancer cell lines (IC_50_ = 0.2–2.7 µM). Overall, the structural diversity of fungal α‐pyrones and the related structure–activity relationships highlight their promise as potential scaffolds for anticancer, antimicrobial, and anti‐inflammatory drug discovery.

### 2.5 Others Compounds

**FIGURE 22:**
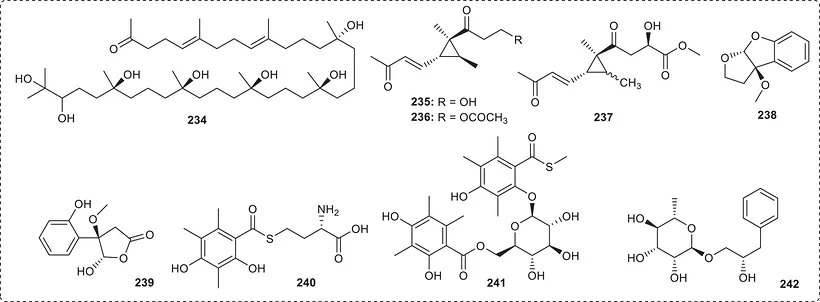
Structures of compounds **234–242**.

A glisoprenin analogue not reported before, glisoprenin G (**234**, Figure 22), was obtained from the fermentation culture of *Gliocladium* sp. F1, an endophytic fungal isolate recovered from the fresh root tissues of the medicinal plant *M. fortunei* collected in Nanjing, China. The cytotoxic effects of compound **234** were assessed using the human cancer cell lines MDA‐MB‐231 and PC‐3. The compound displayed moderate cytotoxic activity, with IC_50_ values of 9.05 and 19.25 µM, respectively [168]. Similarly, the endophytic fungus, *Schizophyllum* sp. QYM‐12, isolated from fresh leaves of the mangrove plant *B. gymnorrhiza*, collected in Guangdong Province, China, was found to produce five previously undescribed secondary metabolites. These comprised three cyclopropane derivatives (**235**–**237**) and two benzofuran derivatives (**238**–**239**) (Figure 22). Their biological activities were evaluated in vitro in an inflammation model based on lipopolysaccharide (LPS)‐stimulated RAW 264.7 macrophages. Compounds **238** and **239** exhibited significant anti‐inflammatory activity, shown by their ability to inhibit interleukin‐6 (IL‐6) production at 10 µM [169]. Three previously undescribed secondary metabolites‐namely an aromatic amino acid, dimorine A (**240**, Figure 22); a suncheonioside derivative, dimoroside A (**241**), and a phenylpropanoid glycoside, dimoroside B (**242**) (Figure 22) were isolated from the endophytic fungus *U. dimorpha* VDG10, associated with the roots of *V. dunalianum* Wight (*Ericaceae*). The in vitro antifungal activities of these compounds were evaluated against five pathogenic fungi: *A. brassicicola, F. graminearum, F.oxysporum, B. cinerea*, and *C. versicolor*. All metabolites (**240‐**‐**242**) (Figure 22) exhibited antifungal activity against all tested pathogens, with MIC values ranging from 15.63 to 62.5 µg/mL [170].

## 3 Conclusions

This systematic review highlights endophytic fungi as an exceptionally rich and versatile source of structurally diverse and biologically active secondary metabolites. Analysis of studies published between 2024 and 2025 revealed the discovery of 242 previously uncharacterized compounds, encompassing major chemical classes, such as terpenoids, alkaloids, nitrogen‐containing compounds, and polyketides. Among these, the genera *Aspergillus, Penicillium, Diaporthe, Talaromyces*, and *Trichoderma* emerged as particularly prolific producers. The wide variety of host plants and ecological habitats from which these fungi were isolated underscores the significant influence of biodiversity on metabolite production. Importantly, the isolated compounds exhibited a broad spectrum of bioactivities, including anti‐inflammatory, antimicrobial, antimicroalgal, and cytotoxic effects, highlighting the considerable pharmaceutical potential of endophytic fungal metabolites. These findings emphasize their value as promising lead structures for drug discovery and development. Overall, continued exploration of endophytic fungi is expected to play a pivotal role in expanding natural product–based drug discovery and in identifying novel therapeutic agents.

### 3.1 Emerging Trends, Challenges, and Future Perspectives

Recent progress in metabolomics, genome mining, and molecular biology has markedly improved the rate at which structurally varied and biologically active secondary metabolites are discovered from endophytic fungi. Still, much of the present work concentrates on isolating individual compounds, clarifying their structures, and performing early‐stage bioactivity screening. Compared with this, studies that address biosynthetic pathways, the underlying molecular mechanisms of action, pharmacokinetic properties, and actual in vivo efficacy are much less common. Moreover, differences in cultivation settings, extraction workflows, and how bioactivity is measured often make it hard to directly compare findings between studies, which can weaken reproducibility and limit how readily results can be translated into practical applications. In future research, it will be important to bring together multi‐omics strategies including genomics, transcriptomics, proteomics, and metabolomics together with genome‐guided approaches to natural product discovery. This combined framework should help identify previously unknown biosynthetic pathways and cryptic metabolites. Techniques such as CRISPR/Cas‐mediated stimulation of silent biosynthetic gene clusters (BGCs), heterologous expression, co‐cultivation, and AI‐assisted metabolite annotation and dereplication are also expected to expand the chemical diversity that can be accessed from endophytic fungi. Among these options, co‐cultivation has proven particularly useful for awakening silent or cryptic BGCs, including polyketide synthase (PKS), nonribosomal peptide synthetase (NRPS), and hybrid PKS–NRPS systems. Because co‐culture models natural microbial interactions, it can drive the production of specialized metabolites that are not formed or cannot be detected when the organisms are grown alone. For example, when *Aspergillus* sp. was grown together with *Penicillium* sp., the production of compound **5** increased. In contrast, compounds **175** and **176** were recovered only from co‐cultures involving *Ascomycota* sp. and *Trichoderma harzianum*, which supports the view that interactions between species can activate cryptic biosynthetic pathways. The metabolites induced under these conditions may improve fungal ecological fitness by serving as chemical defense agents against competing microorganisms and by supporting better adaptation in host‐associated environments. Overall, continued efforts to study less well‐characterized fungal taxa across a variety of ecological niches paired with comprehensive pharmacological, toxicological, and mechanistic evaluations will be critical to turning endophytic fungal metabolites into clinically relevant therapeutics and sustainable biotechnological products. Co‐culture‐based strategies, alongside the growing use of genome‐guided and AI‐enabled methods, offer strong routes for uncovering the hidden biosynthetic capacity of endophytic fungi and for accelerating the discovery of new natural product scaffolds.

## Author Contributions

**Dilmurod Murodullayeva**: Conception and design of the study, acquisition of data, analysis, and interpretation of data. **Jakhongir Movlanov**: Writing‐preparation of the manuscript, revision. **Liu Wei**: Writing‐preparation of the manuscript, revision. **Wei Liu**: Writing‐preparation of the manuscript, revision. **Maksud Isokov**: Writing‐preparation of the manuscript, revision. **Santosh Kumar Bose**: Analysis and interpretation of data. **Fazliddin Kobilov**: Analysis and interpretation of data. **Zamira Jumayeva**: Analysis and interpretation of data. **Buston Islamov**: Writing‐preparation of the manuscript, revision. **Jamila Sherkulova**: Analysis and interpretation of data. **Zafar Matyakubov**: Analysis and interpretation of data. **Muniskhon Meylieva**: Analysis and interpretation of data. **Munisa Ergasheva**: Conception and design of the study, acquisition of data, analysis, and interpretation of data. **Diyor Abduraxmanov**: Analysis and interpretation of data. **Guozheng Huang**: Writing‐conception and design of the study, preparation of the manuscript, revision. **Hongchen Jiang**: Writing‐preparation of the manuscript, revision. **Kakhramon Davranov**: Writing‐conception and design of the study, preparation of the manuscript, revision. **Guohui Pan**: Writing‐conception and design of the study, preparation of the manuscript, revision, and final approval of the submitted version. **Nigora Rustamovaa**: Conception and design of the study, acquisition of data, analysis and interpretation of data, preparation of the manuscript, revision, and final approval of the submitted version.

## Funding

This work was supported by the fundamental research project entitled “Discovery of bio control agents against plant pathogenic microorganisms in agricultural crops, investigation of their mechanisms of action, and development of promising producer strains using advanced technologies”.

## Conflicts of Interest

The authors declare no conflicts of interest.

## Data Availability

Data sharing not applicable to this article as no datasets were generated or analyzed during the current study.
